# Mechanistic insights into the inhibitory effect of theaflavins on virulence factors production in *Streptococcus mutans*

**DOI:** 10.1186/s13568-021-01263-z

**Published:** 2021-07-09

**Authors:** Junhao Kong, Kai Xia, Xiaoqin Su, Xuan Zheng, Chunhua Diao, Xiufang Yang, Xiaobo Zuo, Jun Xu, Xinle Liang

**Affiliations:** 1 School of Food Science and Biotechnology, Zhejiang Gongshang University, Hangzhou, 310018 China; 2 Hangzhou Tea Research Institute, CHINA COOP, Hangzhou, 310016 China; 3 Institute of Food Biotechnology, Zhejiang Gongshang University, Hangzhou, 310018 China; 4 Zhejiang Key Laboratory of Transboundary Applied Technology for Tea Resource, Hangzhou, 310016 China; 5 Department of Biological Sciences, Rensselaer Polytechnic Institute, Troy, NY 12180 USA

**Keywords:** Antibacterial activity, Virulence factors, Oxidative stress, Biofilm formation, Two-component system

## Abstract

*Streptococcus mutans* is the primary etiological agent associated with cariogenic process. The present study aimed to investigate the antibacterial and anti-virulence activities of theaflavins (TFs) to *Streptococcus mutans* UA159 as well as the underlying mechanisms. The results showed that TFs were capable of suppressing the acid production, cell adherence, water-insoluble exopolysaccharides production, and biofilm formation by *S. mutans* UA159 with a dosage-dependent manner while without influencing the cell growth. By a genome-wide transcriptome analysis (RNA-seq), we found that TFs attenuated the biofilm formation of *S. mutans* UA159 by inhibiting glucosyltransferases activity and the production of glucan-binding proteins (GbpB and GbpC) instead of directly blocking the expression of genes coding for glucosyltransferases. Further, TFs inhibited the expression of genes implicated in peptidoglycan synthesis, glycolysis, lipid synthesis, two-component system, signaling peptide transport (*comA*), oxidative stress response, and DNA replication and repair, suggesting that TFs suppressed the virulence factors of *S. mutans* UA159 by affecting the signal transduction and cell envelope stability, and weakening the ability of cells on oxidative stress resistance. In addition, an upregulated expression of the genes involved in protein biosynthesis, amino acid metabolism, and transport system upon TFs treatment indicated that cells increase the protein synthesis and nutrients uptake as one self-protective mechanism to cope with stress caused by TFs. The results of this study increase our current understanding of the anti-virulence activity of TFs on *S. mutans* and provide clues for the use of TFs in the prevention of dental caries.

**Supplementary Information:**

The online version contains supplementary material available at 10.1186/s13568-021-01263-z.

## Key points

TFs exert antibacterial and anti-virulence activities with a dosage-dependent manner.

TFs attenuate virulence factors of *S. mutans* by interfering with signal transduction.

TFs act on *S. mutans* virulence networks with pluralistic regulations.

## Introduction

The human oral cavity is an ideal niche for the inhabitation of diverse microorganisms that constitute a predominant part of the human microbial library (Lamont et al. 2018). Under normal circumstance, the oral microbiota live in symbiosis with the host, contributing substantially to the healthy state by suppressing the proliferation of pathogenic microorganisms (Shang et al. 2020). Nevertheless, the imbalance of oral microbiota caused by factors like antibiotic treatment, sugar consumption, and overexposure to fermentable carbohydrate will lead to the development of oral diseases, such as dental caries and periodontitis, which gives rise to healthy risks (Bowen et al. 2018; Lamont et al. 2018). Dental caries characterized by a decalcification of dental hard tissues is mainly caused by the accumulation of acidogenic and aciduric microorganisms, among which *Streptococcus mutans* is the primary etiological agent as it possesses diverse virulence features (Rather et al. 2020). *S. mutans* is capable of metabolizing the dietary carbohydrates to produce diverse organic acids that induce the demineralization of enamel, resulting in the progression of dental caries (Vijayakumar et al. 2021). Furthermore, the robust ability to form biofilm and adapt to stressful conditions during host colonization enables *S. mutans* to adhere enduringly to the tooth surface and escape from elimination by general oral cleaning and care (Souissi et al. 2021; Priya et al. 2021). Therefore, inhibiting the biofilm formation of *S. mutans* is one important way for preventing dental caries development.

At present, diverse antibacterial agents have been applied for the prevention and treatment of dental caries, such as sodium fluoride, chlorhexidine, and stannous ions (Filho et al. 2020; Anderson et al. 2020). These antibacterial agents when contained in oral care products such as toothpaste and mouth washes have been successfully used to prevent dental caries. However, there are growing concerns about the widespread use of such broad-spectrum antimicrobial agents as they would contribute to the antimicrobial resistance (Zhang et al. 2021; Philip et al. 2018). In addition, the excessive use of fluoride results in adverse effects like fluorosis which limits its wide application (Li et al. 2020). These cases lead to the urgent need of novel agents against *S. mutans*. In contrast to the traditional antibacterial agents, use of the extracts from natural plants has been a preferable strategy for the prevention of dental caries (Singla et al. 2018). As an example, previous studies showed that the extracts from *Pistacia vera* L. oleoresin and berry are capable of inhibiting the production of virulence factors and biofilm in *S. mutans* while not reducing bacterial growth (Magi et al. 2018; Souissi et al. 2021).

Theaflavins (TFs) are the main bioactive component of black tea that is widely consumed around the world (Zhang et al. 2020a, b). At present, the antibacterial, antifungal, and antivirus activities of TFs are well-documented (Betts et al. 2013; Cui et al. 2020; Sato et al. 2020). Nevertheless, the inhibitory effect of TFs on the virulence factors production by *S. mutans* has rarely been investigated. A recent study showed that theaflavin-3, 3-digallat (TF3) treatment could suppress the biofilm formation, acid production, and acid tolerance of *S. mutans* (Wang et al. 2019). By real-time PCR (RT-PCR) analysis, the authors also found that the inhibitory effect of TF3 on *S. mutans* virulence was achieved by repressing the expression of genes related to agmatine deiminase system and two-component systems (TCSs, e.g., LytS/LytT, VicK/VicR, and ComD/ComE). However, it is far from fully understanding the underlying mechanisms explaining the inhibitory influence of TFs on *S. mutans* virulence as only few genes have been tested yet. The current study aimed to assess the inhibitory effect of TFs on the virulence factors of *S. mutans*, including acid production, biofilm formation, bacterial adherence, glucosyltransferases activity, and water-insoluble exopolysaccharides (EPS) production. Moreover, the cellular bioprocesses targeted by TFs were elucidated at the transcriptional level by a genome-wide transcriptome analysis.

## Materials and methods

### Bacteria and culture condition

*Streptococcus mutans* UA159 was purchased from the Guangdong Microbial Culture Collection Center (GDMCC, accession no. GIM1.518). *S. mutans* UA159 was grown in brain heart infusion (BHI) medium (Haibo, Qingdao, China) at 37 °C using an anaerobic incubator (Memmert, Germany). The ratio of O_2_, CO_2_, and N_2_ in the incubator was set as 2:1:17.

### Determination of minimum inhibitory concentration

The theaflavins (with purity 80%) were synthesized by enzymatic reaction, which was performed as previously described (the detailed work flow was shown in ref. Kong et al. 2021b). The TFs were dissolved in methanol to make a stock solution of 100 mg/mL, which was stored at − 20 °C before use. The minimum inhibitory concentration (MIC) of *S. mutans* UA159 to TFs was determined as previously reported. Shortly, a 24-h overnight culture of *S. mutans* UA159 was diluted by 1:100 into fresh BHI medium containing TFs with serially diluted concentrations. The mixtures were added to 96-well plates (200 µL in each well) and incubated at 37 °C anaerobically for 24 h. The optical density (OD_600_) was determined by a microtiter plate reader (Victor™X3, PerkinElmer, Waltham, MA, USA). The lowest TFs concentration that inhibited visible bacterial growth was defined as the MIC (OD_600_ change < 0.05). All experiments were conducted with three biological replicates.

### Assay of the antibacterial activity of TFs

To examine the effect of TFs on the growth of *S. mutans* UA159, a 24-h overnight culture of *S. mutans* UA159 was diluted by 1:100 into 10-mL BHI medium containing TFs with a final concentration of 1-, 0.8-, 0.4-, or 0.2-fold the MIC in 15-mL glass tubes, followed by incubating anaerobically at 37 °C. The fresh medium and methanol (2%, v/v) were used as the negative control (NC) and solvent control (SC), respectively. During the incubation process, the cell growth was monitored at designated time points by measuring the culture density at a wavelength of 600 nm.

The killing curves of TFs towards *S. mutans* UA159 were analyzed as previously reported (Wang et al. 2019), with some modifications. Shortly, a 24-h overnight culture of *S. mutans* UA159 was diluted by 1:100 into 10-mL BHI medium and grown to the exponential-phase (OD_600_ = 0.7 ± 0.05). Thereafter, TFs with a final concentration of 5- and 10-fold the MIC were added, respectively, followed by incubating at 37 °C. The survival ratio of cells was measured every 1 h by counting colony-forming units (CFU/mL). All experiments were conducted with three biological replicates.

### Assay of virulence factors production

The assays of virulence factors including cell adherence, acid production, and water-insoluble EPS production in *S. mutans* UA159 were performed as previously reported (Huang et al. 2020; Chen et al. 2016), with some modifications. For the assay of acid production, cells from a 24-h overnight culture of *S. mutans* UA159 were collected by centrifugation at 4, 500 rpm for 5 min, followed by washing with 1 × PBS (pH 7.2) for two times. Thereafter, the cells were suspended in fresh BHI medium containing sucrose with a final concentration of 10 g/L at a concentration of 1 × 10^7^ CFU/mL. Then, TFs with a final concentration of 0.3, 0.5, 1.0, or 2.0 mg/mL were added and the initial pH of the mixture was adjusted to 7.0 using NaOH (0.5 M), followed by incubating anaerobically at 37 °C for 24 h. During the incubation process, changes of the culture pH were monitored at the designated time points by a pH meter (Mettler-Toledo, Zurich, Switzerland).

For the assay of bacterial adherence, a 24-h overnight culture of *S. mutans* UA159 was diluted by 1:100 into 10-mL BHI medium containing 10 g/L sucrose and TFs with a final concentration of 0.3, 0.5, 1.0, or 2.0 mg/mL in 15-mL glass tubes. The tubes containing medium without TFs and with 2% (v/v) methanol were set as the NC and SC groups, respectively. Afterwards, the cultures were incubated anaerobically at 37 °C at an angle of 30° for 24 h. Then, the planktonic cells suspension was discarded and the adhering cells were rinsed gently using NaOH (0.5 M), followed by suspending the adherent cells using 1× PBS. The adherence was quantified by measuring the cell density (OD_600_), while the results were expressed as the percentage obtained by dividing the values of treated groups by that of the control groups.

For the assay of water-insoluble EPS production, a 24-h overnight culture of *S. mutans* UA159 was diluted by 1:100 into 10-mL BHI medium containing 10 g/L sucrose and TFs with a final concentration of 0.3, 0.5, 1.0, or 2.0 mg/mL in 15-mL glass tubes. After incubation for 24 h, the culture was centrifuged at 12,000 rpm for 10 min and the supernatant was discarded, while the precipitate was washed two times using 5-mL distilled water. Then, the precipitate was suspended in 5-mL NaOH (1.0 M), followed by centrifugation at 12,000 rpm for 10 min to obtain the supernatant for water-insoluble EPS analysis. Afterwards, ethanol (95%, v/v) of 15-mL was added to 5-mL supernatant, followed by storing at 4 °C for 12 h to precipitate the EPS. The EPS were collected by centrifugation at 12,000 rpm for 30 min and dissolved in solution composed of 2.5-mL NaOH (0.5 M) and 2.5-mL distilled water. The quantification of EPS was carried out using the anthrone-sulfuric acid colorimetric method as described previously (Chen et al. 2016).

For the assay of glucosyltransferases activity, a 24-h overnight culture of *S. mutans* UA159 was diluted by 1:100 into 10-mL BHI medium containing 10 g/L sucrose and TFs with a final concentration of 0.3, 0.5, 1.0, or 2.0 mg/mL in 15-mL glass tubes. After incubation for 16 h, the culture supernatants were collected by centrifugation at 16,000 rpm for 15 min and ultrafiltered through a 10 kDa membrane (Merck Millipore, MA, USA), followed by concentration using polyethyleneglycol 20,000 (Merck Millipore, MA, USA) and 3 kDa membrane and dialysis to obtain the crude glycosyltransferases (Xiao et al. 2007). The Bradford method was used to determine the concentration of crude glucosyltransferases (Bradford 1976). The glucosyltransferases activity was determined by incubating glucosyltransferases with sucrose and estimating the amount of water-soluble and insoluble glucans formed using the phenol-sulphuric acid method (Veloz et al. 2016). Enzyme activity was expressed in units/mg protein, in which one unit of glucosyltransferases activity was defined as the amount of the enzyme catalyzing the transfer of 1 µM glucose to glucan per min. The results were shown as percentage obtained by dividing the glucosyltransferases activity of treated groups by that of the control groups. All experiments were performed with three biological replicates.

### Assay of biofilm formation

The influence of TFs on the biofilm formation by *S. mutans* UA159 was investigated by a crystalline violet staining method and observed by electron microscopes, which was performed as previously described with some modifications (Xu et al. 2020; Niu et al. 2020). Briefly, a 24-h overnight culture of *S. mutans* UA159 was diluted by 1:100 into 200-µL BHI medium containing 10 g/L sucrose and TFs with a final concentration of 0.3, 0.5, 1.0, or 2.0 mg/mL in a 96- well polystyrene culture plate (final OD_600_ of ~ 0.05). After incubation for 24 h, the medium was discarded and the wells were gently rinsed with 1 × PBS to remove the planktonic and loosely adhering cells. The adherent biofilm was quantified by staining the cells with 1% crystal violet solution for 30 min at room temperature. The excess crystal violet was removed by washing the wells three times with distilled water. Crystal violet bound to the adherent cells was solubilized in 1-mL 95% ethanol (v/v) and quantified by measuring absorbance at a wavelength of 595 nm.

For the biofilm formation assay using confocal laser scanning microscopy (CLSM), the sterilized glass slides were put into a 24-well polystyrene culture plate before the adding of a 10-mL culture mixture consisting of an overnight culture of *S. mutans* UA159 (1%, v/v), sucrose (10 g/L), and TFs with a final concentration of either 0.5 or 1.0 mg/mL, and the saliva coating was formed on the glass slides in advance. Meanwhile, the untreated culture and 2% (v/v) methanol were used as the NC and SC groups, respectively. After anaerobic incubation at 37 °C for 24 h, the culture supernatants were discarded and the slides were taken out and washed three times using 1 × PBS, followed by staining using SYTO® 9 and propidium iodide nucleic acid stains according to the manufacturer’s instructions (Invitrogen, CA, USA). Then the slides were observed by a confocal laser scanning microscope (Zeiss LSM 880, Oberkochen, Germany) equipped with a 60 × oil immersion objective lens, while five fields-of-view were photographed for each sample.

For the biofilm formation assay using a scanning electron microscope (SEM), the sterilized glass slides were put into a 24-well polystyrene culture plate as described above. After anaerobic incubation at 37 °C for 24 h, the glass slides after washing were first air-dried at room temperature, followed by fixing overnight in PBS solution (pH 7.4) containing 2.5% (v/v) glutaraldehyde and 2% (v/v) formaldehyde at 4 °C. Afterwards, the slides were washed twice using 1 × PBS, and then dehydrated in solutions with increasing concentrations of ethanol (35, 50, and 75% for 30 min each and two cycles of 90 and 100% for 30 min each) (Niu et al. 2020). Thereafter, the slide samples were dried, sputter coated with gold, and observed under a scanning electron microscope (Hitachi Regulus 8100, Tokyo, Japan).

### Transcriptome analysis

A genome-wide transcriptional response of *S. mutans* UA159 to TFs was investigated by RNA-sequencing (RNA-seq) analysis, which was performed as previously described (the detailed work flow was shown in ref. Xia et al. 2020). Briefly, a 24-overnight culture of *S. mutans* UA159 was diluted by 1:100 into 100-mL fresh BHI medium containing 10 g/L sucrose and TFs with a final concentration of 0.5 mg/mL in 250-mL Erlenmeyer flasks. After anaerobic incubation for 24 h, the cells were collected by centrifugation at 12,000 rpm under 4 °C for 10 min. Cell samples collected from three independent cultures with TFs or without TFs were used for the transcriptome analysis (the samples were named as X2 and X1, respectively), which was performed by Biozeron Biotechnology Co., Ltd. (Jiading, Shanghai, China). Total RNA was extracted using TRIzol® Reagent according the manufacturer’s instructions (Invitrogen, Carlsbad, CA, USA), and the genomic DNA was removed using DNase I (TaKaRa, Changping, Beijing, China). Then the RNA quality was determined using a 2100 Bioanalyzer (Agilent, Santa Clara, CA, USA) and quantified using the ND-2000 (NanoDrop Technologies, Wilmington, USA). RNA-seq strand-specific libraries were prepared following TruSeq RNA sample preparation Kit from Illumina (San Diego, CA, USA), using 5 µg of total RNA. cDNA synthesis, end repair, A-base addition, and ligation of the Illumina-indexed adaptors were performed according to the Illumina’s protocol. Libraries were then size selected for cDNA target fragments of 200–300 bp on 2% low range ultra-agarose, followed by PCR amplification using Phusion DNA polymerase (NEB, Ipswich, MA, USA) for 15 PCR cycles. After quantified by TBS380 (Picogreen, Invitrogen, USA), paired-end libraries were sequenced using the Illumina NovaSeq 6000 with 2 × 150 bp read length.

The raw paired-end reads were trimmed and quality controlled by Trimmomatic (version 0.36) with default parameters (SLIDINGWINDOW: 4:15; MINLEN: 75) (Bolger et al. 2014). Then the clean reads were separately aligned to the reference genome (accession no. NC_004350.2) with orientation mode using the Rockhopper software (Tjaden 2015). EdgeR was used for the differential expression analysis (Robinson et al. 2010). The differentially expressed genes (DEGs) were selected using the following criteria: |Log_2_FC| ≥ 1, the fold change (FC) was calculated by dividing the expression level (RPKM, the fragments per kilobase of read per million mapped reads) of each gene in X2 by that in X1, while the false discovery rate (FDR) should be less than 0.05. To understand the function of the DEGs, GO functional enrichment and KEGG pathway analyses were carried out by GOATOOLS (Klopfenstein et al. 2018) and KOBAS (Xie et al. 2011), respectively. DEGs were significantly enriched in GO terms and metabolic pathways when their Bonferroni-corrected *p* value was less than 0.05.

### Statistical analysis

All statistical analyses were performed using the Origin software (version 9.0) (OriginLab, Northampton, MA, USA). Where appropriate, the data were analyzed using the Student’s t test and a one-way analysis of variance (ANOVA) with a Bonferroni’s multiple-comparison test. Differences were considered statistically significant at *p* < 0.05. Values were shown as the mean of three biological replicates ± standard deviation.

## Results

### The inhibitory effect of TFs on the growth of *S. mutans* UA159

**Fig. 1:**
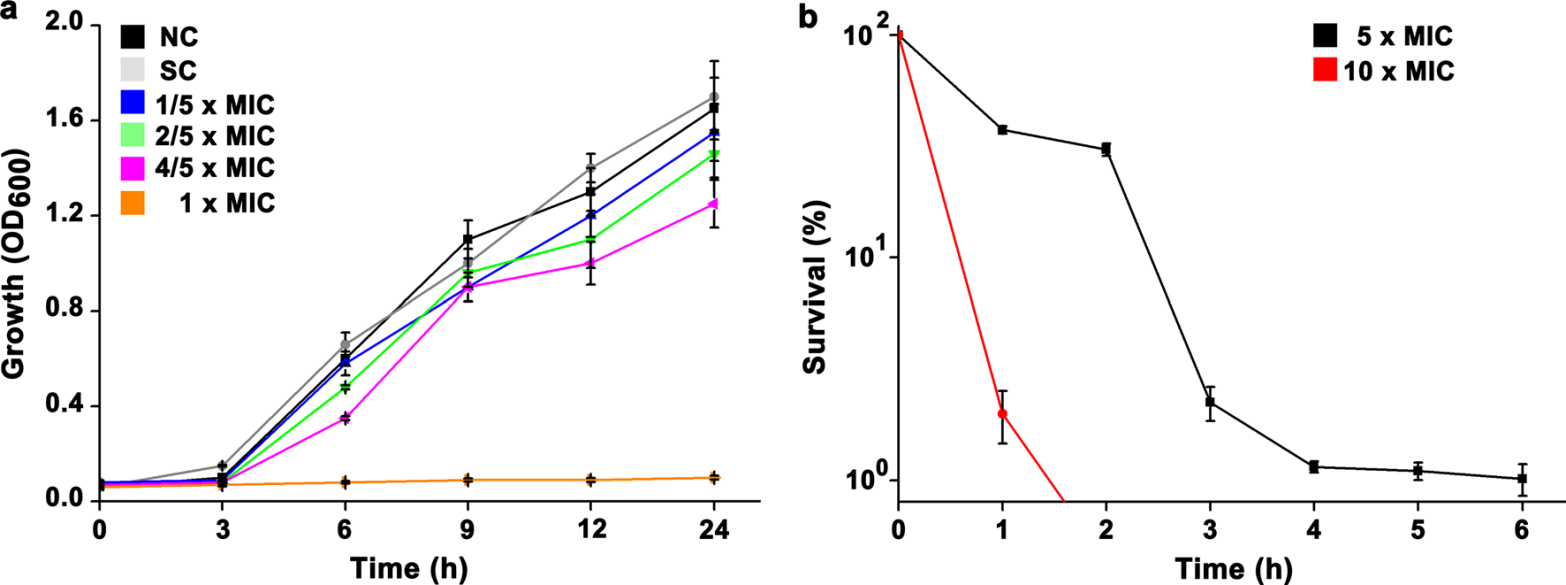
The inhibitory effect of TFs on the growth of *S. mutans* UA159. **a** The growth curves of *S. mutans* UA159 in medium containing TFs with concentrations below the MIC. **b** the killing curves of *S. mutans* UA159 upon TFs treatment with concentrations higher than the MIC. All experiments were conducted with three biological replicates, and error bars represent standard deviations. NC and SC indicate negative control and solvent control, respectively

By the bioassay, the MIC of *S. mutans* UA159 to TFs was determined by 2.5 mg/mL. To investigate the inhibitory effect of TFs with sub-MIC on the growth of *S. mutans* UA159, cells were grown in medium containing 0.5 (1/5 × MIC), 1.0 (2/5 × MIC), or 2.0 mg/mL (4/5 × MIC) TFs for 24 h. The results showed that the growth curves of cells treated by either 0.5 or 1.0 mg/mL TFs were not significantly different compared with that in the control groups (NC and SC) (Fig. 1a). Treatment using TFs with a final concentration of 2.0 mg/mL inhibited the cell growth within 9 h, after which the inhibitory effect was not significant. To investigate the bactericidal effect of TFs on *S. mutans* UA159 at concentrations higher than the MIC, the exponential-phase cultures were treated by TFs with a final concentration of 5- and 10-fold the MIC, respectively. We found that the bulk of bacterial population was rapidly killed by TFs of 5 × MIC within 4 h, leaving a plateau of surviving cells with survival ratio around 1% (99% killing) (Fig. 1b). Moreover, all cells were killed within 2 h during the treatment of TFs with a concentration of 10 × MIC. Collectively, these results suggested that the antibacterial activity of TFs on *S. mutans* UA159 is dosage-dependent.

### TFs inhibited the virulence factors production by *S. mutans* UA159

**Fig. 2:**
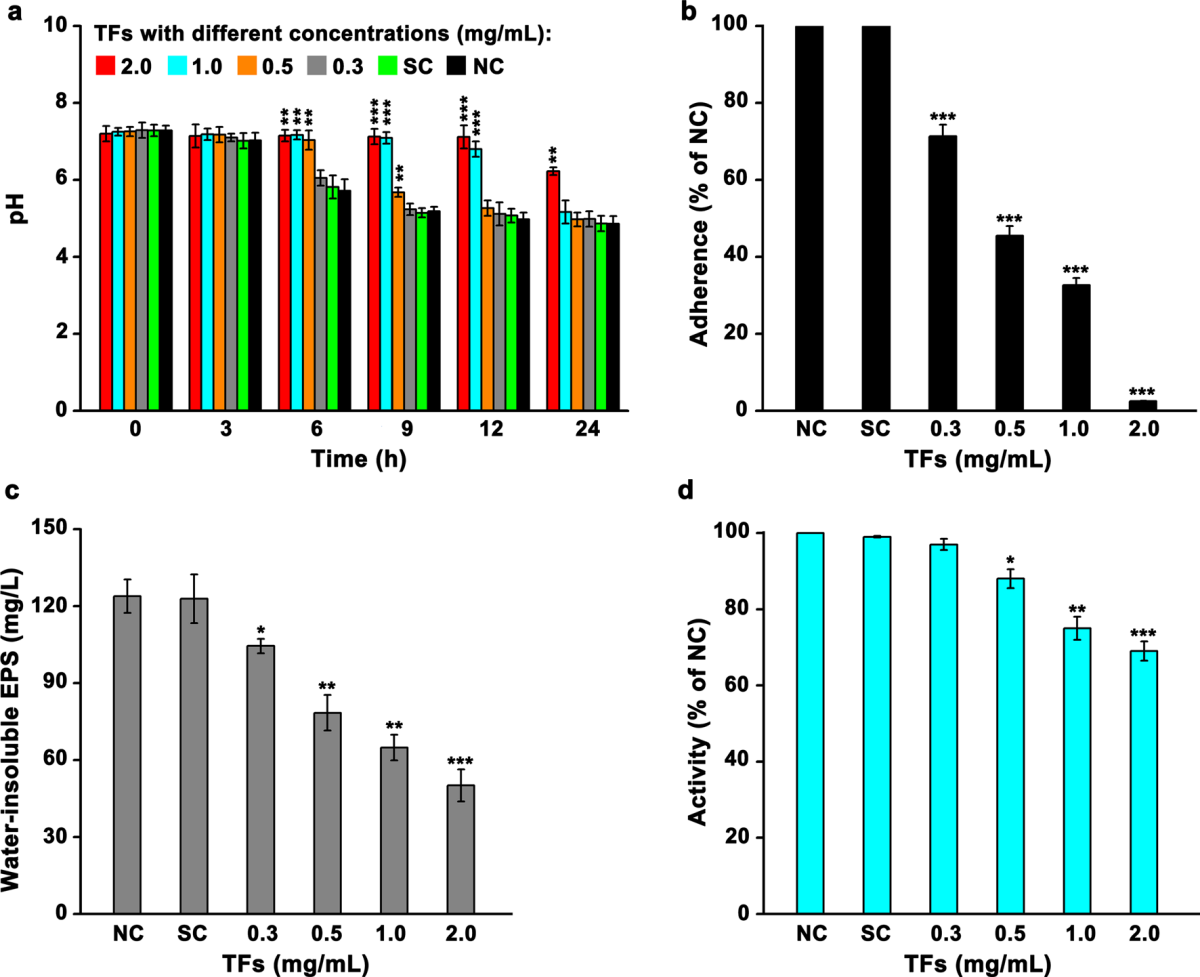
Assay of the virulence factors production by *S. mutans* UA159. The inhibitory effect of TFs on the acid production (**a**), adherence (**b**), water-insoluble EPS production (**c**), and glucosyltransferases activity (**d**). Data obtained from three biological replicates were shown as mean ± standard deviation. “*” represents that there is a significant difference between two studied groups (*p < 0.05; **p < 0.01; ***p < 0.001). The difference was compared between the group with TFs added or the SC and the NC group. In (**a**), the difference between two groups was compared within the same time points. NC and SC indicate negative control and solvent control, respectively

The inhibitory effect of TFs with concentrations below the MIC on the virulence factors including acid production, water-insoluble EPS production, and cell adherence in *S. mutans* UA159 was investigated. The results showed that the acid production of *S. mutans* UA159 grown in the medium containing TFs with a final concentration of 0.5, 1.0, or 2.0 mg/mL was significantly inhibited within 6 h compared with that of the control groups (Fig. 2a). After incubation for 9 h, the pH of medium without TFs or with TFs of 0.3 or 0.5 mg/mL dropped, while such trend was not observed in medium with TFs of 1.0 mg/mL and 2.0 mg/mL. At the end of the growth, the pH of medium containing 2.0 mg/mL TFs was significantly higher than that in other groups. Similar to the inhibitory effect of TFs on acid production, TFs with a sub-MIC also significantly decreased the cell adherence with a dosage-dependent manner (Fig. 2b). With 0.3 mg/mL TFs treatment, an overall 35% decrease in adherence was observed. The cell adherence dropped to only 45 and 30% of the NC when 0.5 and 1.0 mg/mL TFs were used, respectively. With 2.0 mg/mL TFs treatment, the cell adherence dropped to 2.5% of the NC. Meanwhile, treatment with sub-MIC TFs also significantly suppressed the EPS production, wherein the EPS concentration gradually decreased as the TFs concentration in the medium increased (Fig. 2c). The decreased production of EPS upon TFs treatment might result from the decrease of glucosyltransferases activity (Fig. 2d). With 1.0 or 2.0 mg/mL TFs treatment, the glucosyltransferases activity dropped to an overall 65% of that in the control group (NC).

### TFs inhibited biofilm formation by *S. mutans* UA159

**Fig. 3:**
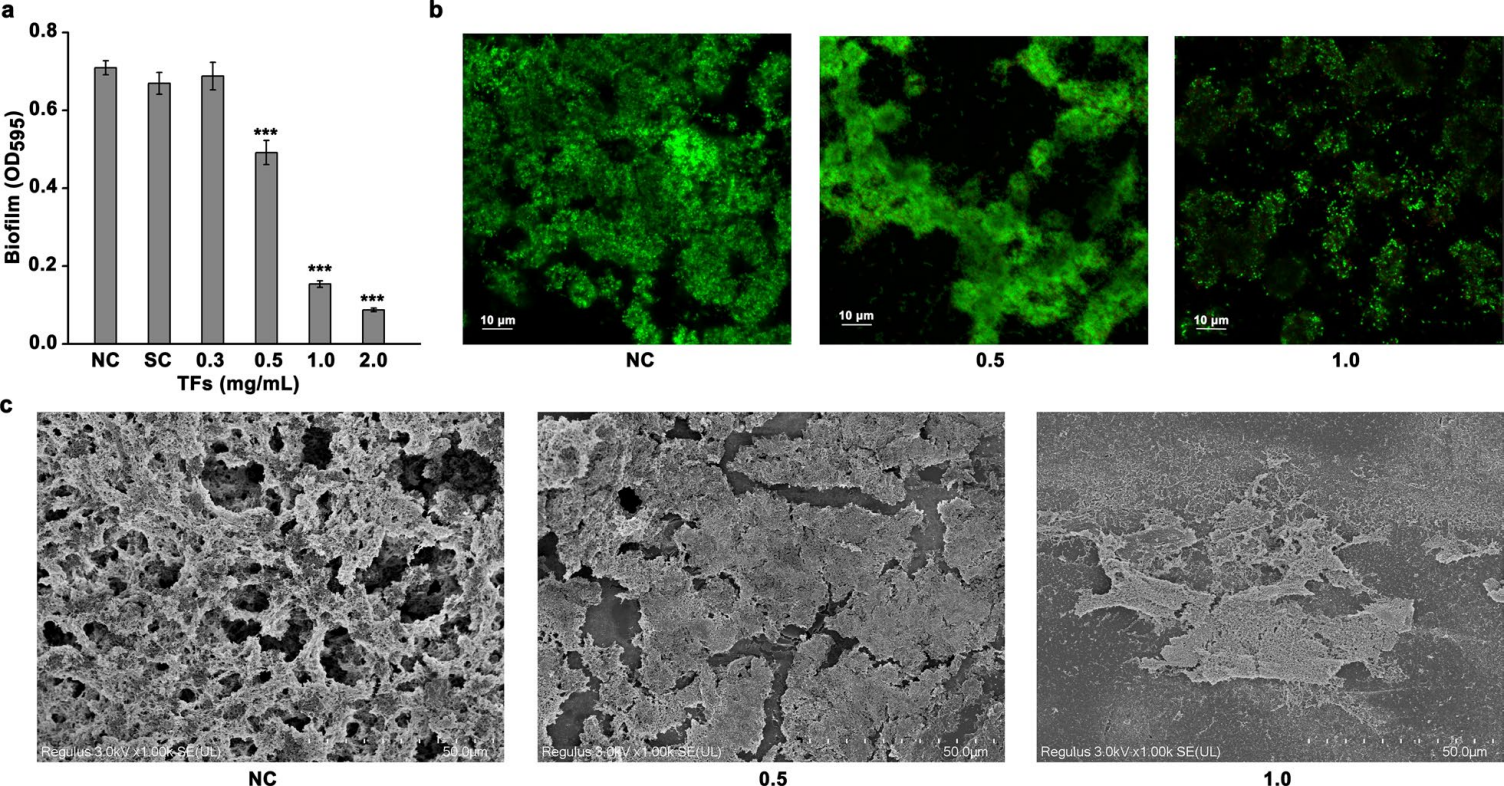
Assay of the biofilm formation by *S. mutans* UA159. **a** Inhibitory effect of TFs on the biofilm formation quantified by crystal violet staining assay. Data obtained from three biological replicates were shown as mean ± standard deviation. “*” represents that there is a significant difference between two studied groups (***p < 0.001). The difference was compared between the group with TFs added and the NC. Representative images showing the inhibitory effect of TFs on the biofilm formation by using confocal laser scanning microscopy (**b**) and a scanning electron microscope (**c**). The live and dead cells could be stained by SYTO® 9 and propidium iodide (PI) nucleic acid stains, respectively

By a crystal violet staining assay, we found that TFs were capable of inhibiting the biofilm formation by *S. mutans* UA159 (Fig. 3a). Treatment using TFs of 0.5 mg/mL significantly suppressed the biofilm formation by at least 30% compared with that in the NC, while an overall reduction of 80 and 90% in the biofilm formation were observed by 1.0 and 2.0 mg/mL TFs, respectively. At a low concentration of 0.3 mg/mL, TFs had no significant inhibitory effect on the biofilm formation. Moreover, the adding of 2% methanol (solvent control) did not significantly inhibited the biofilm formation compared with that in the NC group.

By imaging using confocal laser scanning microscopy, we found a clear reduction in the *S. mutans* UA159 biofilms in the presence of TFs with concentrations of 0.5 and 1.0 mg/mL as assessed by the live/dead cell staining (Fig. 3b). In comparison with the control group that the biofilms were thick and mostly green, TFs treatment decreased the biofilm thickness and formation as the green regions decreased, which supported the results obtained by crystal staining assay. Further, the formed biofilm was observed by a scanning electron microscope. Compared with the control group (NC) in which a thick and uniform biofilm was formed after a 24-h incubation, the addition of TFs with a final concentration of either 0.5 or 1.0 mg/mL significantly attenuated the biofilm formation and produced an obviously thinned sheet-like biofilm (Fig. 3c).

### The influence of TFs on global gene expression in *S. mutans* UA159

**Fig. 4:**
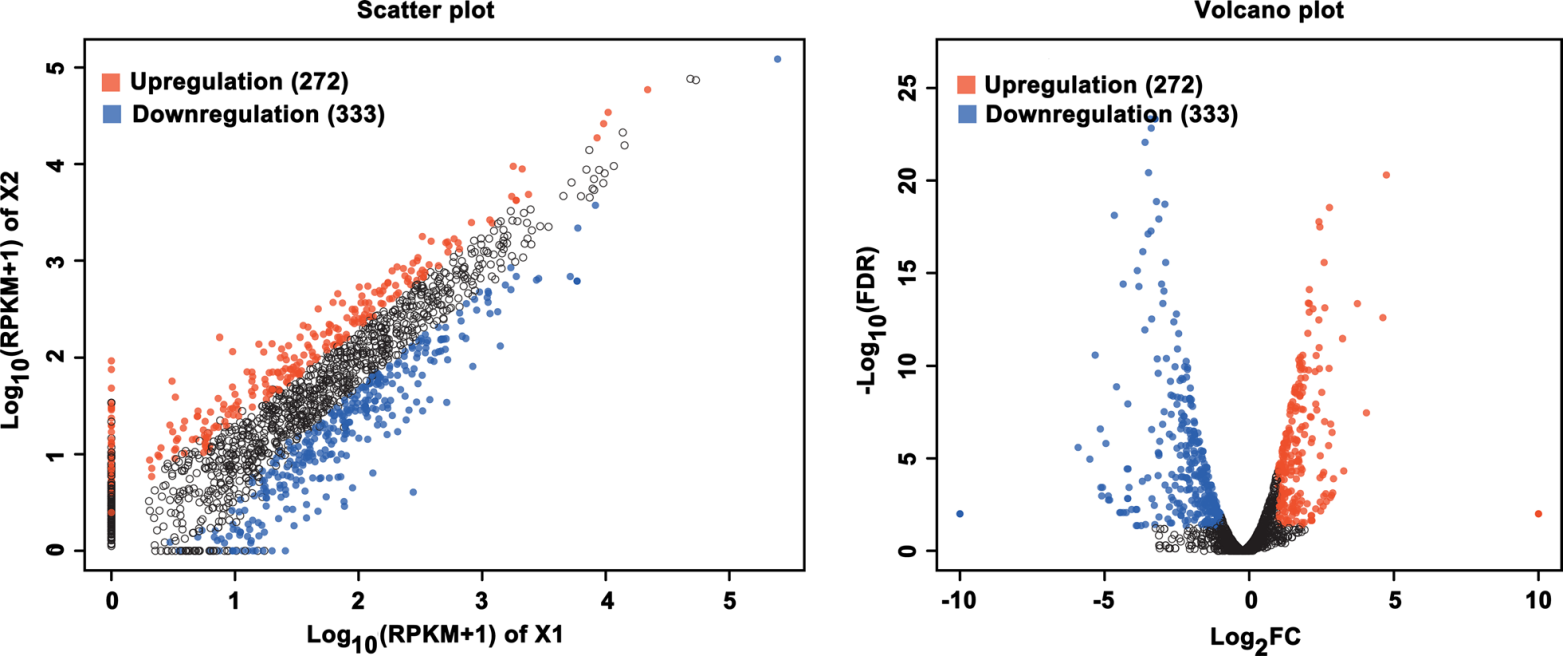
Visualized distribution of the differentially expressed genes between X2 and X1 by scatter plot and volcano plot

**Fig. 5:**
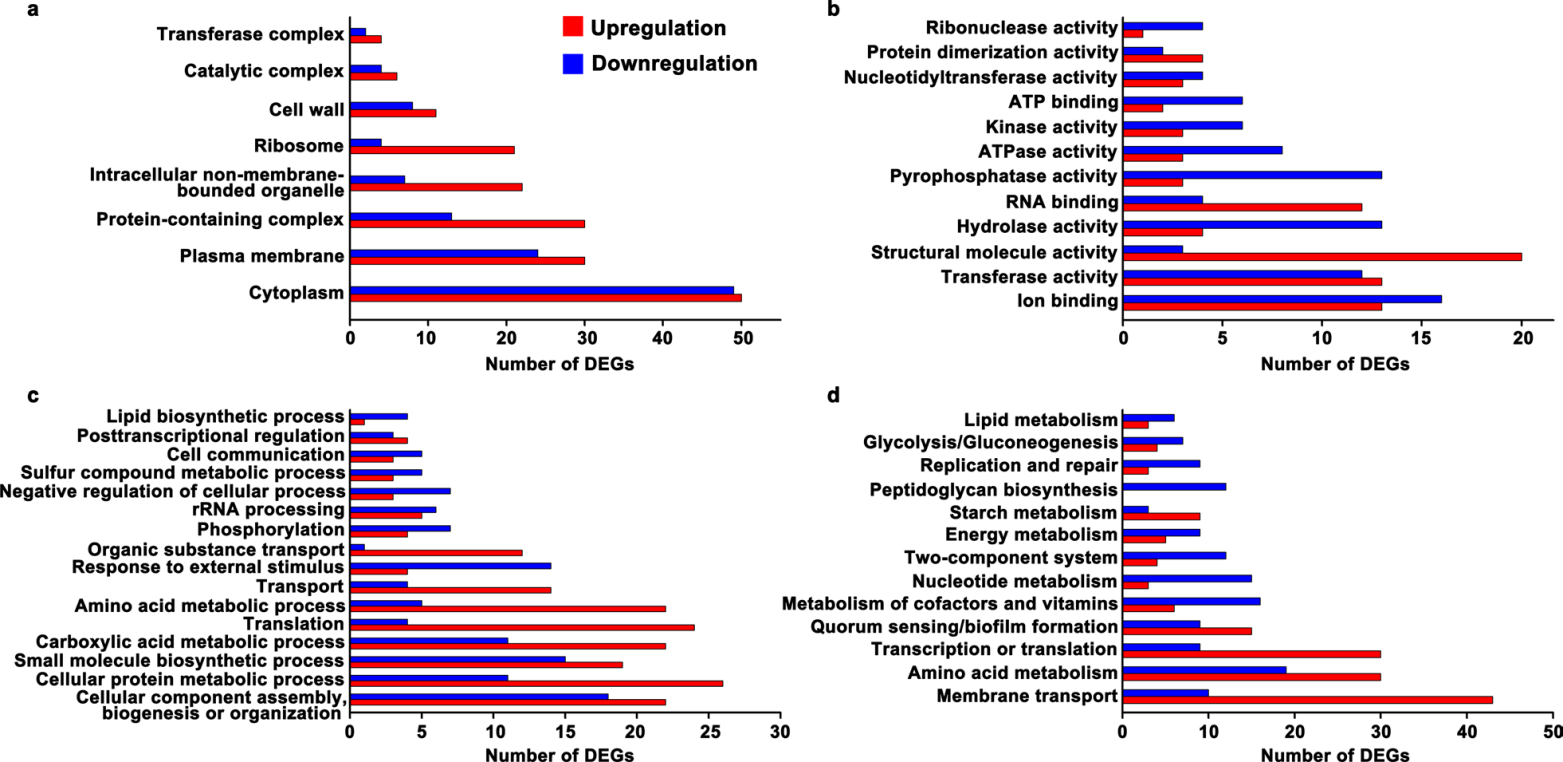
GO and KEGG enrichment cluster analyses of the differentially expressed genes. The DEGs related to cellular component (**a**), molecular function (**b**), and biological process (**c**). **d** KEGG pathway cluster of the differential genes

After knowing the inhibitory effect of TFs on the virulence factors production by *S. mutans* UA159 while without influencing the cell growth, we tried to unravel the underlying molecular mechanisms. To achieve this, we compared a genome-wide transcriptional response of the cells grown in medium containing TFs with a final concentration of 0.5 mg/mL with that grown in the medium without TFs. Gene expression analysis identified 605 DEGs in X2 compared with that in X1 (|Log_2_FC| >1; FDR < 0.05), of which 272 genes were shown to be upregulated (Fig. 4). One hundred and seventy six genes encoding hypothetical proteins were not discussed in this study. GO functional enrichment analysis showed that most of the DEGs encode proteins that locate mainly in cytoplasm, plasma membrane, and protein-containing complex (Fig. 5a). These proteins predominantly possess ion-binding activity, transferase activity, structural molecule activity, hydrolase activity, and RNA-binding activity, participating in biological processes like cellular component assembly, biogenesis or organization, cellular protein metabolic process, small molecule biosynthetic process, carboxylic acid metabolic process, and translation (Fig. 5b and c). KEGG enrichment analysis presented that these DEGs were mainly implicated in membrane transport, amino acid metabolism, transcription or translation, quorum sensing or biofilm formation, metabolism of cofactors and vitamins, and two-component system (Fig. 5d). We next conducted a detailed study of the transcriptional changes of these important pathways and within the functional groups revealed by the enrichment analysis.

### DEGs involved in cell envelope elements

**Fig. 6:**
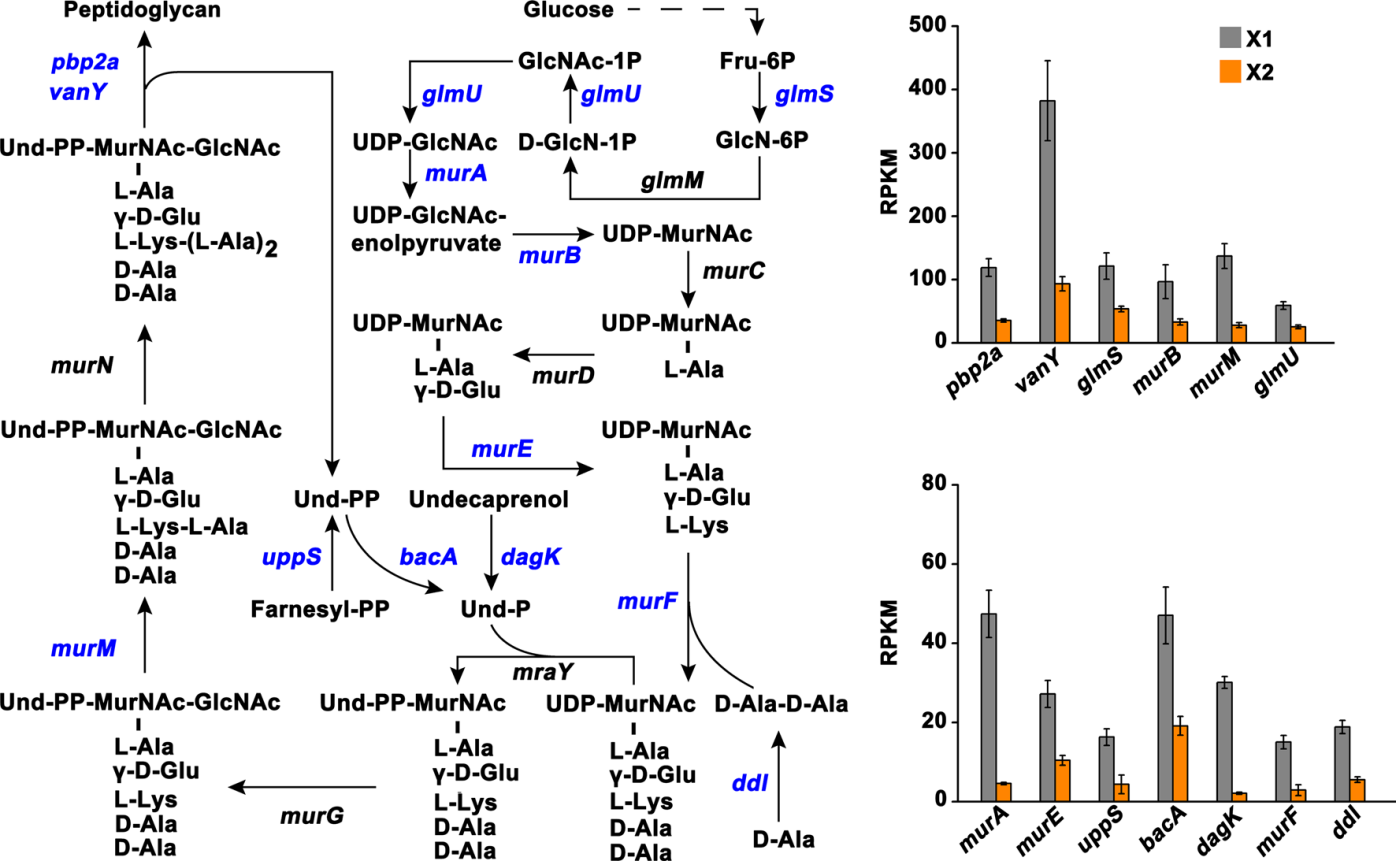
Changes in the expression of genes involved in peptidoglycan biosynthesis. Genes colored with blue indicate downregulation. Expression level shown as RPKM was displayed by mean ± standard deviation. Dashed arrow indicates multiple steps. The detailed information of these DEGs were presented in Additional file 1: Table S1

**Fig. 7:**
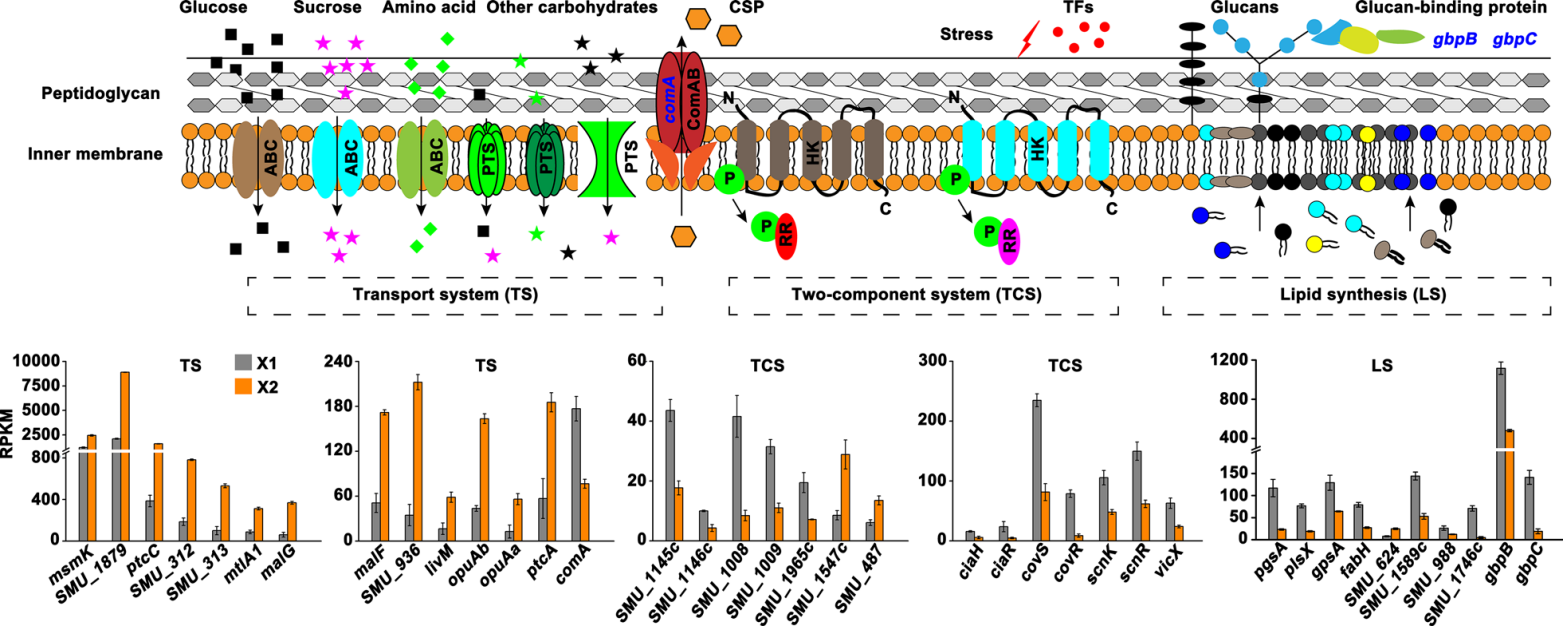
Changes in the expression of genes involved in transport system, two-component system, and lipid synthesis. Genes colored with blue indicate downregulation. Only part of the genes with a relatively higher fold change were displayed. Expression level shown as RPKM was displayed by mean ± standard deviation. The detailed information of these DEGs were presented in Additional file 1: Table S1. ABC: ATP-binding cassette; PTS: sugar phosphotransferase system; HK: histidine kinase; RR: response regulator; P in green circle indicates phosphoryl group; CSP: competence-stimulating peptide

Notably, 121 genes related to cell envelope elements including ATP-binding cassette (ABC) transporter, sugar phosphotransferase system (PTS), peptidoglycan, membrane lipid, and two-component system (TCS) were found to be differentially expressed upon TFs treatment, among which 58 and 63 genes were downregulated and upregulated, respectively (Additional file 1: Table S1). For the peptidoglycan biosynthesis, we observed that TFs treatment repressed the expression of 13 genes (*pbp2a*, *vanY*, *murM*, *glmU*, *glmS*, *murA*, *murB*, *murE*, *uppS*, *bacA*, *dagK*, *murF*, and *ddl*) included in the biosynthesis process (Fig. 6), with fold changes ranging from 0.07 to 0.50 (Additional file 1: Table S1). The expression of most of the genes coding for ABC transporters (45 out of 60 genes) and PTS transporters (14 out of 19 genes) was induced upon TFs treatment, with fold changes ranging from 2.02 to 7.41. These transporters were mainly responsible for the transport of sugars (e.g., *msmK*, *SMU_1879*, *ptcC*, *SMU_312*, *SMU_313*, *mtlA1*, *malG, malF*, and *ptcA*) and amino acids (e.g., *SMU_936*, *livM*, *opuAa*, and *opuAb*) (Fig. 7 and Additional file 1: Table S1). For the downregulated transporters, it was noteworthy for a gene *comA* that encodes an ABC transporter involved in the transport of competence-stimulating peptide (CSP). In addition, the expression of 7 genes implicated in lipid synthesis, including phosphotidylglycerophosphate synthase (*pgsA*), glycerol-3-phosphate dehydrogenase (*gpsA*), cardiolipin synthase (*SMU_988*), hexosyltransferase (*SMU_1589c*), fatty acid/phospholipid synthesis protein (*plsX*), 3-oxoacyl-[acyl-carrier-protein] synthase III (*fabH*), and enoyl-CoA hydratase (*SMU_1746c*), was repressed in X2 compared with that in X1.

Similar to the observations that TFs inhibited the expression of genes involved in peptidoglycan and lipid synthesis, the mRNA levels of genes coding for the histidine kinases (*ciaH*, *SMU_1145c*, *covS*, *scnK*, *SMU_1965c*, and *SMU_1009*) and response regulators (*ciaR, SMU_1146c, covR*, *vicX*, *scnR*, *SMU_1008*, *SMU_1547c*, and *SMU_487*) that were involved in signal transduction were almost all significantly decreased in X2 compared with that in X1 (Fig. 7). In addition, two genes coding for glucan-binding protein C (*gbpC*) and secreted antigen GbpB/SagA (*gbpB*) that were related to biofilm formation were downregulated upon TFs treatment (Additional file 1: Table S1). Collectively, these findings suggested that TFs have role in interfering with nutrients uptake and signal transduction, and impacting the cell envelope stability of *S. mutans* UA159.

### DEGs involved in glycolysis

**Fig. 8:**
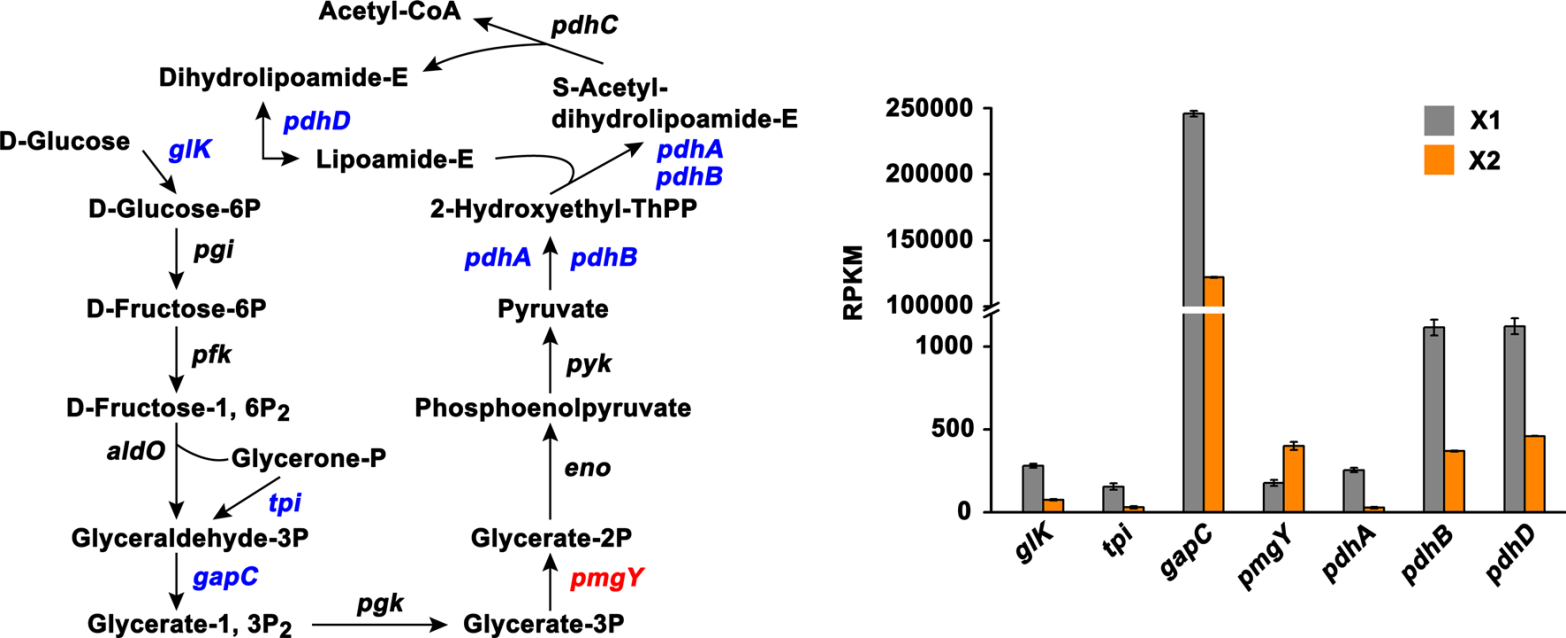
Changes in the expression of genes implicated in glycolysis. The upregulated and downregulated genes were denoted as red and blue texts, respectively. Expression level shown as RPKM was displayed by mean ± standard deviation. The detailed information of these DEGs were presented in Additional file 1: Table S2

Glycolysis plays an important role in the acid production of *S. mutans* UA159, contributing to the development of dental caries. It was worth noting that TFs treatment significantly decreased the mRNA levels of genes encoding glucose kinase (*glk*), triosephosphate isomerase (*tpi*), glyceraldehyde-3-phosphate dehydrogenase (*gapC*), acetoin dehydrogenase (*pdhA* and *pdhB*), and dihydrolipoamide dehydrogenase (*pdhD*) that were involved in glycolysis (Fig. 8 and Additional file 1: Table S2), suggesting that TFs decreased the acid production of *S. mutans* by inhibiting glycolysis.

### DEGs involved in protein homeostasis and amino acid metabolism

**Fig. 9:**
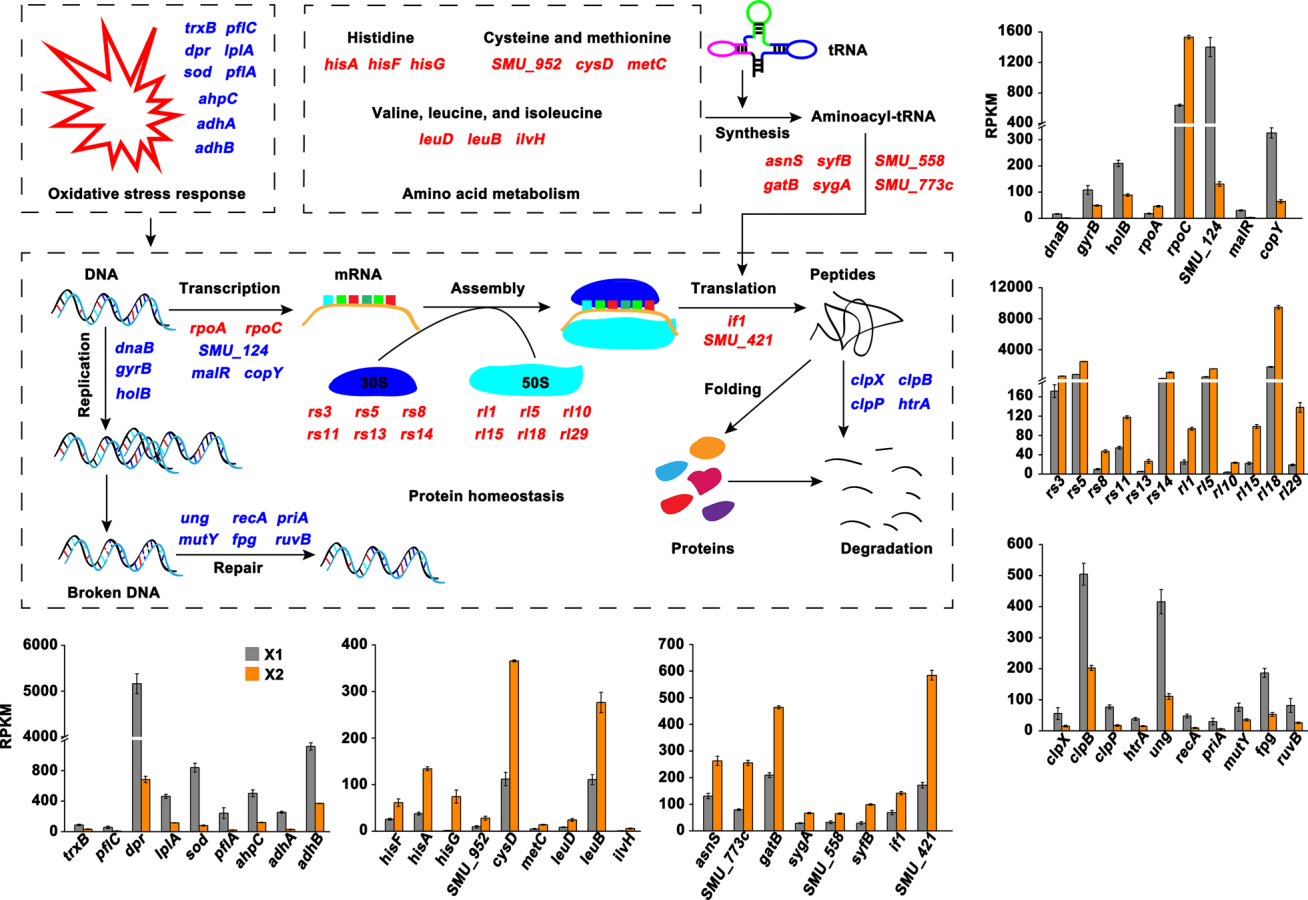
Changes in the expression of genes implicated in protein homeostasis, amino acid metabolism, DNA replication, repair, and recombination, and oxidative stress response. The upregulated and downregulated genes were denoted as red and blue texts, respectively. Expression level shown as RPKM was displayed by mean ± standard deviation. The detailed information of these DEGs were presented in Additional file 1: Tables S3 and S4

Totally, 99 genes related to protein homeostasis and amino acid metabolism were differentially expressed in X2 compared with that in X1 (Additional file 1: Table S3), among which the expression of 62 genes was induced. For the protein homeostasis, the upregulated genes mainly encode proteins implicated in the translation process, such as the large and small ribosomal proteins (e.g., *rs3*, *rs5*, *rs8*, *rs11*, *rs13*, *rs14*, *rl1*, *rl5*, *rl10*, *rl15*, *rl18*, and *rl29*) and translation factors (*if1* and *SMU_421*), and the aminoacyl-tRNA synthesis process (e.g., *asnS*, *syfB*, *SMU_558*, *gatB*, *SMU_773c*, and *sygA*) (Fig. 9). Furthermore, the expression of genes coding for ATP-dependent protease Clp (*clpX*, *clpP*, and *clpB*) and serine protease HtrA (*htrA*) was repressed upon TFs treatment. These findings suggested that cells increase the protein synthesis while decrease the protein degradation to resist stress caused by TFs. In addition, it was interesting to find that the mRNA levels of 11 out of 15 genes coding for transcriptional regulators (e.g., *SMU_124*, *malR*, and *copY*) were significantly decreased in X2 compared with that in X1 while the mRNA levels of genes encoding DNA-dependent RNA polymerase (*rpoA* and *rpoC*) were increased, which indicated a dynamical regulation of the transcription process (Fig. 9).

Functional enrichment analysis indicated that TFs treatment induced amino acid metabolism. We found that among the 38 DEGs involved in amino acid metabolism, 25 genes were significantly upregulated in X2 compared with that in X1 (Additional file 1: Table S3). Specifically, the upregulated genes were mainly implicated in the metabolism of histidine (e.g., *hisA*, *hisF*, and *hisG*), and cysteine and methionine (e.g., *cysD*, *SMU_952*, and *metC*) (Fig. 9). Notably, the expression of genes coding for 3-isopropylmalate dehydratase (*leuD*), 3-isopropylmalate dehydrogenase (*leuB*), and acetolactate synthase (*ilvH*) that were related to the biosynthesis of valine, leucine, and isoleucine was induced, which indicated that cells increase the synthesis of branched-chain amino acids to cope with stress caused by TFs.

### DEGs involved in DNA replication, recombination, repair, and oxidative stress response

Functional enrichment analysis of the DEGs suggested that TFs have role in suppressing the DNA replication and oxidative stress response of *S. mutans* UA159. We found that the expression of genes coding for DNA polymerase III (*dnaN* and *holB*), chromosome replication protein (*dnaB*), and DNA gyrase (*gyrA* and *gyrB*) that were involved in DNA replication was significantly repressed in X2 compared with that in X1 (Fig. 9 and Additional file 1: Table S4). Similarly, the expression of genes encoding thioredoxin reductase (NADPH) (*trxB*), peroxide resistance protein Dpr (*dpr*), manganese-type superoxide dismutase (*sod*), alkyl hydroperoxide reductase (*ahpC*), acetoin dehydrogenase (*adhA* and *adhB*), lipoate-protein ligase (*lplA*), and pyruvate-formate lyase activating enzyme (*pflA* and *pflC*) that were responsible for the oxidative stress response was downregulated in X2 compared with that in X1. In addition, among the 11 DEGs involved in DNA repair and recombination, 9 genes coding for uracil DNA glycosylase (*ung*), formamidopyrimidine-DNA glycosylase (*fpg*), A/G-specific DNA glycosylase (*mutY*), 3-methyl-adenine DNA glycosylase I (*tagI*), recombination protein RecA (*recA*), RecF protein (*recF*), primosomal replication factor Y (*priA*), holliday junction DNA helicase RuvB (*ruvB*), and endonuclease (*SMU_1485c*) were shown to be downregulated upon TFs treatment.

## Discussion

### TFs exert antibacterial and anti-virulence activities with a dosage-dependent manner

The growing concerns of the side effects produced by the use of traditional antibacterial agents such as chlorhexidine and fluoride for the prevention of dental caries caused by *S. mutans* has shifted the public attention to natural products (Singla et al. 2018; Souissi et al. 2021; Vijayakumar et al. 2021). As an example, baicalein, a flavonoid isolated from the roots of *Scutellaria baicalensis* and *Scutellaria lateriflora*, is capable of inhibiting the biofilm formation, acid production, and cell surface hydrophobicity in *S. mutans* while without inhibiting its growth (Vijayakumar et al. 2021). Theaflavins are the main bioactive component of black tea, which mainly contains four monomers including theaflavin (TF1), theaflavin-3-gallate (TF2a), theaflavin-3′-gallate (TF2b), and theaflavin-3, 3′-digallate (TF3) (Zhang et al. 2020a, b). At present, the antibacterial activity of TFs on microorganisms such as *Acinetobacter baumannii*, *Stenotrophomonas maltophilia*, and *Bacillus coagulans* is clear (Betts et al. 2011; Sato et al. 2020). However, the inhibitory effect of TFs on *S. mutans* has not yet been fully investigated. In a recent study, Wang and co-workers showed that TF3 suppresses the biofilm formation, acid production, and acid tolerance by *S. mutans* UA159 (Wang et al. 2019). In line with their findings, we found that TFs exert antibacterial and anti-virulence activities towards *S. mutans* UA159 with a dosage-dependent manner. For one thing, TFs with sub-MIC did not inhibit the cell growth significantly while a concentration higher than the MIC could kill the cells rapidly. For another, TFs with sub-MIC were able to inhibit the biofilm formation, acid production, cell adherence, and EPS production by *S. mutans* UA159, while the inhibitory effect of TFs was dependent on the concentrations used. In our previous work, we revealed that tooth brushing using toothpaste containing 2.0 mg/mL TFs is able to reduce the oral pathogenic bacteria (e.g., *Prevotella*, *Selenomonas*, and *Atopobium*) while increased the abundance of oral-health associated bacteria (e.g., *Streptococcus* and *Rothia*) in healthy adults (Kong et al. 2021b). Collectively, these findings would facilitate the use of oral health products containing TFs for the prevention of dental caries formation.

### TFs inhibit the virulence of *S. mutans* UA159 by interfering with signal transduction

Bacteria have evolved sophisticated systems to sense and adapt to the dynamically changed environments. Two-component system (TCS), consisting of a sensory histidine kinase and a response regulator, is one kind of such systems involved in sensing environmental cues and regulating adaptive responses (Tiwari et al. 2020; Xia et al. 2020). In *S. mutans*, there is abundant evidence showing that the virulence factors production is co-regulated by diverse TCSs, and 15 TCSs have been found in the genome of *S. mutans* (Shanmugam et al. 2020). For example, a quorum-sensing dependent TCS ComD/ComE is involved in the biofilm formation by mediating the release of chromosomal DNA into the extracellular matrix, while a ComD/ComE deficient strain produces biofilm with substantially reduced biomass (Li et al. 2002). Another TCS ScnK/ScnR is shown to be implicated in the formation of a sponge-like biofilm architecture and play an important role in defending against oxidative stress (Chen et al. 2008; Shanmugam et al. 2020). In addition, TCSs like VicK/VicR, CiaH/CiaR, and LiaS/LiaR are reveled to contribute to the acid tolerance, oxidative stress response, and competence of *S. mutans* (Liu and Burne 2009; Gong et al. 2009). Under this background, result that the inhibitory effect of natural product on the virulence of *S. mutans* is achieved by repressing the expression of diverse TCSs has been widely reported (Rocha et al. 2020). As an example, *Rhodiola rosea* extract repressed the expression of ComD/ComE, eventually leading to the decreased biofilm formation of *S. mutans* (Zhang et al. 2020a, b). In a former study, Wang and co-workers showed that TF3 inhibits the virulence factors production by *S. mutans* UA159 by blocking the expression of genes coding for LytS/LytT, ComD/ComE, and VicK/VicR TCSs (Wang et al. 2019). In this study, however, the expression of these three TCSs was not found to be significantly repressed upon the TFs treatment; instead, TFs treatment inhibited the expression of another three known TCSs CiaH/CiaR, ScnK/ScnR, and CovS/CovR (Fig. 7). The difference might result from the different experimental conditions, TFs compositions, and TFs concentrations used in two studies. On the other hand, TFs treatment also inhibited the expression of another two TCSs SMU_1145c/SMU_1146c and SMU_1009/ SMU_1008. The functions and regulatory networks of these two TCSs are not known yet, which needs future investigation. Furthermore, although the expression of QS-based TCS ComD/ComE was not significantly affected by TFs, TFs treatment repressed the expression of *comA* coding for an ABC transporter responsible for the efflux of signaling peptide CSP that is critical for the ComD/ComE to carry out its regulatory roles after activation in the virulence factors production by *S. mutans* (Rocha et al. 2020). Collectively, our findings suggest that the inhibitory effect of TFs on the virulence of *S. mutans* UA159 is achieved partly by repressing the expression of TCSs that are involved in signal transduction.

### TFs inhibit the virulence of *S. mutans* UA159 by a combination of multiple regulations

Polysaccharide, extracellular DNA (eDNA), and adhesin proteins are the main components of *S. mutans* biofilm (Shanmugam et al. 2020). It is known that the *gtf* clusters (*gtfB*, *gtfC*, and *gtfD*) and *gbp* clusters (*gbpA*, *gbpB*, *gbpC*, and *gbpD*) coding for the glucosyltransferases responsible for glucan production and the glucan-binding proteins, respectively, are indispensable for the biofilm formation of *S. mutans* (Mishra et al. 2015). Previous study showed that TF3 was capable of reducing the glucan synthesis by repressing the expression of *gtfB*, *gtfC*, and *gtfD* (Wang et al. 2019). Moreover, TF3 treatment decreased the mRNA levels of *lrgA*, *lrgB*, and *srtA* that were involved in eDNA production by governing cell autolysis and membrane vesicle components. In this study, however, the above described genes were not found to be differentially expressed upon TFs treatment. Nevertheless, we found that TFs treatment decreased the glucosyltransferases activity and the expression of genes related to DNA replication, suggesting that TFs attenuate the biofilm formation of *S. mutans* UA159 by inhibiting the glucosyltransferases activity and DNA synthesis. Meanwhile, how TFs inhibit the glucosyltransferases activity is still unclear. Possibly, TFs may have role in interfering with the binding of glucosyltransferases and substrate by playing as substrate antagonists, which needs future investigation. In addition, we observed that TFs treatment significantly repressed the expression of *gbpB* and *gbpC* (Fig. 7). It has been established that GbpB is essential for the initial phase of sucrose-dependent biofilm formation in *S. mutans* and has influence on the cell shape and cell wall maintenance (Fujita et al. 2007; Duque et al. 2011). GbpC is an important cell-surface protein involved in the adherence and dextran-dependent aggregation of *S. mutans*, contributing to the sucrose-dependent biofilm formation (Biswas et al. 2007; Zhu et al. 2009). On the other hand, we found that TFs treatment significantly repressed the expression of genes related to the synthesis of lipid and peptidoglycan, suggesting that TFs have role in affecting the cell envelope stability. Collectively, these findings suggest that affecting the cell envelope stability, glucosyltransferases activity, and glucan-binding proteins production is one important way for TFs to attenuate the biofilm formation and adherence of *S. mutans*.

The results of this study suggest that TFs have role in weakening the ability of *S. mutans* on oxidative stress resistance. First, TFs repressed the expression of genes directly related to the oxidative stress response. It is known that genes coding for superoxide dismutase (*sod*) and peroxide resistance protein (*dpr*) are indispensable for the oxidative stress tolerance of *S. mutans* (Galvao et al. 2015). An upregulated expression of gene coding for alkyl hydroperoxide reductase (*ahpC*) was found when *S. mutans* biofilms were treated with gentamicin, vancomycin, and linezolid antibiotics that are capable of inducing oxidative stress (Nilsson et al. 2019). Recent studies provided evidence showing that pyruvate as well as the genes implicated in pyruvate metabolism such as *adhABCD* cluster and *pflC* contribute substantially to the oxidative stress tolerance of *S. mutans* (Kajfasz et al. 2017; Ahn et al. 2019). Notably, the expression of all of these genes as well as the genes involved in pyruvate synthesis was repressed upon TFs treatment. Second, TFs repressed the expression of gens involved in DNA repair, including *fpg*, *mutY*, and *recA.* DNA repair systems are critical for *S. mutans* to combat with oxidative stress by maintaining the genome stability (Shanmugam et al. 2020). The genes *fpg* (formamidopyrimidine-DNA glycosylase) and *mutY* (A/G-specific DNA glycosylase) belonging to the base excision repair system are responsible for repairing the oxidized bases (Gonzalez et al. 2012). The gene *recA* involved in SOS repair system induces the repair of damaged DNA caused by reactive oxygen species. Third, TFs induced the protein synthesis. The compromised ability of *S. mutans* on oxidative stress tolerance will inevitably affect the protein synthesis and function since oxidative damage will result in protein misfolding and fragmentation (Kong et al. 2021a). It is worth noting that this case occurs in this study. For one thing, most of the genes implicated in protein synthesis were found to be upregulated while the genes related to protein degradation were downregulated, suggesting an urgent need of protein synthesis as a self-protective mechanism for the cells to cope with stress induced by TFs. For another, the expression of genes related to the transport and metabolism of amino acids was induced, especially the genes involved in the biosynthesis of branched amino acids (isoleucine, leucine, and valine). Previous studies suggested that the increased level of branched amino acids is an indication of destructed protein synthesis (Zhou et al. 2019; Chen et al. 2017). Taken together, these findings suggest that compromising the ability of *S. mutans* on oxidative stress resistance is another important way for TFs to inhibit its virulence factors production.

Last but not the least, it should be pointed out that the way used by TFs to affect the expression of genes related to the above described cellular processes is still elusive. A previous study proved that TF3 inhibits the growth of Zika virus by directly binding to the NS2B-3 protease (ZIKVpro) responsible for the production of NS2B and NS3 proteins that are essential for viral replication (Cui et al. 2020). Another study showed that the interaction between membrane proteins and TF3 is the main factor that determines the antibacterial activity of TF3 towards *B. coagulans*, wherein the ABC transporters are the primary targets (Sato et al. 2020). Recently, by molecular docking analysis, a strong binding affinity was found between VicR or GtfC and usnic acid, resulting in the repressed expression of *vicR*, *gtfC*, and other virulence genes responsible for the decreased production of virulence factors in *S. mutans* UA159 upon usnic acid treatment (Priya et al. 2021). Taking these cases into consideration, it is reasonable to assume that TFs are likely to bind the membrane proteins such TCS modules and other transcriptional regulators through which to affect the expression of genes involved in different virulence pathways, ultimately leading to the impaired virulence of *S. mutans* UA159, which requires further study.

In conclusion, the present study investigated the antibacterial and anti-virulence activities of TFs towards the dental caries-associated bacteria *S. mutans* UA159. TFs with concentrations below the MIC are able to inhibit the virulence factors production by *S. mutans* UA159 with a dosage-dependent manner while without influencing the cell growth. The results of transcriptome analysis suggest that the inhibitory effect of TFs on the virulence of *S. mutans* UA159 is achieved by affecting the signal transduction, cell envelope stability, and glycolysis, and weakening the ability of cells on oxidative stress resistance by repressing the DNA repair system and perturbing protein homeostasis. Nevertheless, how TFs affect the expression of genes involved in these critical cellular processes needs future investigation.

## Supplementary Information

**Additional file 1. Table S1.** Detailed information of DEGs involved in cell envelope elements. **Table S2.** Detailed information of DEGs involved in glycolysis. **Table S3.** Detailed information of DEGs involved in protein homeostasis and amino acid metabolism. **Table S4.** Detailed information of DEGs involved in DNA replication, repair, recombination, and oxidative stress response.

## Data Availability

The RNA-seq experiment results have been submitted to the Sequence Read Archive (SRA) under accession number PRJNA715090.
