# Supplementary material for: Mechanistic insights into the inhibitory effect of theaflavins on virulence factors production in Streptococcus mutans
Source: AMB Express. 2021 Jul 9;11:102. doi: 10.1186/s13568-021-01263-z (PMC8271058; doi:10.1186/s13568-021-01263-z)
Supplement: Supplementary file 1 — Additional file 1. Table S1. Detailed information of DEGs involved in cell envelope elements. Table S2. Detailed information of DEGs involved in glycolysis. Table S3. Detailed information of DEGs involved in protein homeostasis and amino acid metabolism. Table S4. Detailed information of DEGs involved in DNA replication, repair, recombination, and oxidative stress response. [file 13568_2021_1263_MOESM1_ESM.pdf]

AMB Express

Mechanistic insights into the inhibitory effect of theaflavins on virulence factors production in *Streptococcus mutans*

Junhao Kong[1, 2, 4#], Kai Xia[2, 5#*], Xiaoqin Su[2, 4#], Xuan Zheng[1, 3], Chunhua Diao[2, 4], Xiufang Yang[2, 4], Xiaobo Zuo[2, 4], Jun Xu[1, 3], Xinle Liang[1, 3*]

[1]School of Food Science and Biotechnology, Zhejiang Gongshang University, Hangzhou 310018, China

[2]Hangzhou Tea Research Institute, CHINA COOP, Hangzhou 310016, China

[3]Institute of Food Biotechnology, Zhejiang Gongshang University, Hangzhou 310018, China

[4]Zhejiang Key Laboratory of Transboundary Applied Technology for Tea Resource, Hangzhou 310016, China

[5]Department of Biological Sciences, Rensselaer Polytechnic Institute, Troy, New York 12180, USA

[*]Corresponding author:

Xinle Liang, dbiot@mail.zjgsu.edu.cn

Kai Xia, xiakai333@126.com

[#]These authors contributed equally to this work.

Table S1 Detailed information of DEGs involved in cell envelope elements

Gene name	Gene name of UA159	Product	RPKM of X1	RPKM of X2	Fold change
oppB	*SMU_256*	ABC transporter, permease OppB	92.74	290.01	3.13
oppC	*SMU_257*	ABC transporter, permease OppC	92.40	312.42	3.38
oppF	*SMU_259*	ABC transporter, ATP-binding protein OppF	251.31	652.21	2.59
comA	*SMU_286*	Putative ABC transporter, ATP-binding protein ComA	176.85	76.58	0.43
	SMU_431	Putative ABC transporter, ATP-binding protein	5.92	0.62	0.10
	SMU_432	Putative ABC transporter, integral membrane protein	69.47	25.35	0.36
	SMU_524	Putative ABC transporter, ATP-binding protein	12.95	4.74	0.37
	SMU_525	Putative ABC transporter, ATP-binding protein	1.12	6.14	5.38
	SMU_806c	Putative glutamine ABC transporter, permease protein	146.15	487.49	3.34
msmK	*SMU_882*	Multiple sugar-binding ABC transporter, ATP-binding protein, MsmK	1206.80	2445.35	2.03
	SMU_934	Putative amino acid ABC transporter, permease protein	149.04	451.84	3.03
	SMU_936	Putative amino acid ABC transporter, ATP-binding protein	34.66	212.11	6.12
potC	*SMU_975*	Putative spermidine/putrescine ABC transporter, permease protein	27.56	74.45	2.70
potD	*SMU_976*	Putative ABC transporter, periplasmic spermidine/putrescine-binding protein	12.82	29.77	2.32
opuAb	*SMU_1062*	Putative ABC transporter, proline/glycine betaine permease protein	43.55	163.43	3.75
opuAa	*SMU_1063*	Putative ABC transporter, ATP-binding protein	12.71	56.05	4.40
opuBa	*SMU_1096*	Putative ABC transporter, ATP-binding protein, choline transporter	16.91	36.01	2.13
	SMU_1118c	Putative ABC sugar transporter, permease protein	62.88	135.90	2.16
pstS	*SMU_1138*	Putative ABC transporter, phosphate-binding protein	53.37	14.41	0.27
	SMU_1216c	Putative amino acid ABC transporter, permease protein	271.06	124.34	0.46
msmF	*SMU_879*	Multiple sugar-binding ABC transporter, permease protein MsmF	121.37	266.23	2.19
msmG	*SMU_880*	Multiple sugar-binding ABC transporter, permease protein MsmG	262.48	563.49	2.15
adcA	*SMU_1302*	AdcA protein-like protein	1.04	7.70	7.41
glnQ	*SMU_1519*	Putative amino acid ABC transporter, ATP-binding protein	509.60	154.98	0.30
malF	*SMU_1569*	Putative maltose/maltodextrin ABC transporter, permease protein MalF	50.98	171.88	3.37
malG	*SMU_1570*	Putative maltose/maltodextrin ABC transporter, MalG permease	61.15	368.21	6.02

	SMU_1571	Putative ABC transporter, ATP-binding protein, MsmK-like protein	98.97	250.31	2.53
livM	*SMU_1667*	Putative branched chain amino acid ABC transporter, permease protein	16.47	58.62	3.56
sloB	*SMU_183*	Putative Mn/Zn ABC transporter	185.69	404.34	2.18
	SMU_1933c	Possible cobalt permease	35.07	105.39	3.00
	SMU_1934c	Putative cobalt ABC transporter, ATP-binding protein	66.80	160.40	2.40
	SMU_1938c	Putative ABC transporter, permease protein	9.71	23.84	2.46
	SMU_1939c	Putative ABC transporter, ATP-binding protein	21.79	44.87	2.06
atmB	*SMU_1941*	Putative membrane lipoprotein	4.72	12.10	2.56
adcB	*SMU_1993*	Putative ABC transporter, zinc permease protein	9.20	31.33	3.40
opuCc	*SMU_2118*	Glycine betaine/carnitine/choline ABC transporter	156.58	355.17	2.27
opuCd	*SMU_2119*	Putative osmoprotectant ABC transporter; permease protein	371.33	905.69	2.44
	SMU_247	Putative ABC transporter, ATP-binding protein	161.88	34.87	0.22
	SMU_413	Putative ABC transporter, ATP-binding protein	39.09	11.45	0.29
	SMU_459	Putative ABC transporter, amino acid binding protein	4.80	13.32	2.78
	SMU_568	Putative amino acid ABC transporter, ATP-binding protein	13.49	47.97	3.55
	SMU_651c	Putative ABC transporter, substrate-binding protein	156.14	484.38	3.10
	SMU_652c	Putative ABC transporter, ATP-binding protein	120.06	364.74	3.04
	SMU_653c	Putative ABC transporter, permease protein	58.37	212.64	3.64
	SMU_805c	Putative amino acid ABC transporter, ATP-binding protein	592.17	1681.70	2.84
	SMU_827	Putative polysaccharide ABC transporter, permease protein	7.28	20.78	2.85
	SMU_863	Putative ABC transporter, ATP-binding protein	21.16	69.75	3.30
glrA	*SMU_1035*	Putative ABC transporter, ATP-binding protein	2.26	8.01	3.55
	SMU_1068c	Putative ABC transporter, ATP-binding protein	236.85	38.90	0.16
	SMU_1078c	Putative ABC transporter, ATP-binding protein	19.17	69.93	3.65
	SMU_1079c	Putative ABC transporter, ATP-binding protein	12.90	62.60	4.85
	SMU_1166c	Putative ABC transporter, permease protein	5.11	10.26	2.01
	SMU_1217c	Putative ABC transporter, amino acid binding protein	30.18	68.45	2.27
	SMU_1348c	Putative ABC transporter, ATP-binding protein	134.98	34.54	0.26

	SMU_1366c	Putative ABC transporter, ATP-binding protein	134.98	34.54	0.26
psaB	*SMU_1928*	Putative ABC transporter, permease protein	5.76	17.83	3.09
adcC	*SMU_1994*	Putative ABC transporter, ATP-binding protein	28.82	10.58	0.37
	SMU_2159	Putative ABC transporter, ATP-binding protein	461.54	151.54	0.33
	SMU_923	Putative ABC transporter, ATP-binding protein	8506.55	18688.81	2.20
	SMU_1898	Putative ABC transporter, ATP-binding and permease protein	23.91	3.81	0.16
	SMU_815	Putative amino acid transporter, amino acid-binding protein	334.93	675.04	2.02
	SMU_817	Putative amino acid transporter, amino acid-binding protein	6.73	0.33	0.05
sgaT	*SMU_270*	Putative PTS system, membrane component	538.40	1427.08	2.65
	SMU_311	PTS system, sorbitol (glucitol) phosphotransferase enzyme IIC2	56.72	179.06	3.16
	SMU_312	PTS system, sorbitol phosphotransferase enzyme IIBC	185.89	782.45	4.21
	SMU_313	Putative PTS system, sorbitol-specific enzyme IIA	100.31	531.92	5.30
mtlA1	*SMU_1185*	PTS system, mannitol-specific enzyme IIBC component	87.86	312.88	3.56
lacF	*SMU_1492*	PTS system, lactose-specific enzyme IIA EIIA-LAC)	25.16	1.70	0.07
ptcC	*SMU_1596*	Putative PTS system, cellobiose-specific IIC component	385.49	1588.81	4.12
ptcA	*SMU_1598*	Putative PTS system, cellobiose-specific IIA component	56.79	185.40	3.26
ptnA	*SMU_1877*	Putative PTS system, mannose-specific component IIAB	523.47	1562.61	2.98
ptnC	*SMU_1878*	Putative PTS system, mannose-specific component IIC	10453.53	34279.94	3.28
	SMU_1879	Putative PTS system, mannose-specific component IID	2107.39	8893.23	4.22
ptsG	*SMU_2047*	Putative PTS system, glucose-specific IIABC component	1152.20	2638.31	2.29
	SMU_100	Putative sorbose PTS system, IIB component	14.45	0.96	0.07
	SMU_101	Putative sorbose PTS system, IIC component	67.25	4.78	0.07
	SMU_102	Putative PTS system, IID component	93.77	29.63	0.32
	SMU_103	Putative PTS system, IIA component	67.29	16.21	0.24
	SMU_115	Putative PTS system, fructose-specific IIA component	26.13	131.97	5.05
	SMU_1957	Putative PTS system, mannose-specific IID component	229.52	827.69	3.61
	SMU_1958c	Putative PTS system, mannose-specific IIC component	327.23	1777.13	5.43
	SMU_263	Putative amino acid antiporter	527.65	1225.48	2.32

ciaH	*SMU_1128*	Putative histidine kinase sensor CiaH	15.71	5.70	0.36
ciaR	*SMU_1129*	Putative response regulator CiaR	23.80	4.67	0.20
	SMU_1145c	Putative histidine kinase	43.56	17.69	0.41
	SMU_1146c	Putative response regulator	10.33	4.29	0.42
covS	*SMU_1516*	Putative histidine kinase CovS; VicK-like protein	234.77	81.38	0.35
covR	*SMU_1517*	Putative response regulator CovR; VicR-like protein	78.67	8.68	0.11
vicX	*SMU_1515*	Conserved hypothetical protein CovX (VicX)	62.91	24.01	0..38
scnK	*SMU_1814*	Putative histidine kinase, ScnK-like protein	105.40	48.10	0.46
scnR	*SMU_1815*	Putative response regulator; ScnR-like protein	149.75	61.80	0.41
	SMU_1965c	Putative histidine kinase	19.46	7.17	0.37
	SMU_1008	Putative response regulator	41.57	8.46	0.20
	SMU_1009	Putative histidine kinase	31.46	11.01	0.35
	SMU_1547c	Putative response regulator	8.57	28.86	3.37
	SMU_487	Putative response regulator	6.11	13.52	2.21
glmS	*SMU_1187*	Glucosamine-fructose-6-phosphate aminotransferase	121.34	53.63	0.44
glmM		Phosphoglucosamine mutase			
glmU	*SMU_1635*	Putative UDP-N-acetylglucosamine pyrophosphorylase	58.97	25.52	0.43
mraY		Phospho-N-acetylmuramoyl-pentapeptide-transferase			
murG		UDP-N-acetylglucosamine-N-acetylmuramyl-(pentapeptide) pyrophosphoryl-undecaprenol N-acetylglucosamine transferase			
murD		UDP-N-acetylmuramoylalanine-D-glutamate ligase			
murC		UDP-N-acetylmuramate--alanine ligase			
vanY	*SMU_75*	Putative D-alanyl-D-alanine carboxypeptidase	382.10	93.21	0.24
murB	*SMU_972*	Putative UDP-N-acetylenolpyruvoylglucosamine reductase	96.57	32.96	0.34
ddl	*SMU_599*	Putative D-alanine-D-alanine ligase	18.87	5.56	0.29
murF	*SMU_603*	Putative D-Ala-D-Ala adding enzyme	15.05	2.91	0.19
	SMU_609	Putative 40K cell wall protein precursor	950.48	472.45	0.50
murM	*SMU_717*	Putative peptidoglycan branched peptide synthesis protein MurM	137.11	28.06	0.20

murN		Alanine adding enzyme			
murA	*SMU_1525*	Putative UDP-N-acetylglucosamine 1-carboxyvinyltransferase	47.43	4.58	0.10
dagK	*SMU_1618*	Diacylglycerol kinase	9.44	0.65	0.07
murE	*SMU_1677*	UDP-MurNac-tripeptide synthetase	27.21	10.46	0.38
bacA	*SMU_1702c*	Putative phosphatase	47.06	19.14	0.41
uppS	*SMU_1786*	Putative undecaprenyl pyrophosphate synthetase	16.30	4.38	0.27
pbp2a	*SMU_1949*	Putative membrane carboxypeptidase, penicillin-binding protein 2a	118.92	35.38	0.30
pgsA	*SMU_2151*	Putative phosphotidylglycerophosphate synthase	116.94	23.36	0.20
gpsA	*SMU_323*	Putative glycerol-3-phosphate dehydrogenase	129.09	64.24	0.50
	SMU_624	Putative 1-acylglycerol-3-phosphate O-acyltransferase	7.79	24.74	3.18
	SMU_988	Putative cardiolipin synthase	26.23	12.32	0.47
	SMU_1589c	Putative hexosyltransferase	144.18	52.82	0.37
plsX	*SMU_26*	Putative fatty acid/phospholipid synthesis protein	76.64	18.96	0.25
fabH	*SMU_1744*	Putative 3-oxoacyl-[acyl-carrier-protein] synthase III	79.10	27.21	0.34
	SMU_1746c	Putative enoyl-CoA hydratase	70.81	4.85	0.07
epsC	*SMU_1437*	Putative UDP-N-acetylglucosamine 2-epimerase	78.59	15.22	0.19
cysE	*SMU_157*	Serine O-acetyltransferase	128.09	63.86	0.50
gbpC	*SMU_1396*	Glucan-binding protein C, GbpC	141.18	19.06	0.14
gbpB	*SMU_22*	Putative secreted antigen GbpB/SagA	1116.14	479.89	0.43

Table S2 Detailed information of DEGs involved in glycolysis

Gene name	Gene name of UA159	Product	RPKM of X1	RPKM of X2	Fold change
pdhA	*SMU_127*	Putative acetoin dehydrogenase (TPP-dependent), E1 component alpha subunit	254.85	27.61	0.11
pdhB	*SMU_128*	Putative acetoin dehydrogenase, E1 component beta subunit	1116.35	369.46	0.33
gapC	*SMU_360*	Extracellular glyceraldehyde-3-phosphate dehydrogenase	245830.50	122089.60	0.50
glk	*SMU_542*	Putative glucose kinase	280.21	75.59	0.27
pmgY	*SMU_596*	Phosphoglyceromutase	177.12	400.79	2.26
tpi	*SMU_715*	Triosephosphate isomerase	155.45	29.58	0.19
pgi		Glucose-6-phosphate isomerase			
pfk		6-Phosphofructokinase			
aldO		Fructose-bisphosphate aldolase			
pgk		Phosphoglycerate kinase			
eno		Enolase			
pyk		Pyruvate kinase			
pdhD	*SMU_1424*	Putative dihydrolipoamide dehydrogenase	1123.70	460.36	0.41
pdhC		Pyruvate dehydrogenase E2 component			

Table S3 Detailed information of DEGs involved in protein homeostasis and amino acid metabolism

Gene name	Gene name of UA159	Product	RPKM of X1	RPKM of X2	Fold change
rpoC	*SMU_1989*	DNA-dependent RNA polymerase, beta' subunit	638.27	1532.71	2.40
rpoA	*SMU_2001*	DNA-directed RNA polymerase, alpha subunit	18.06	46.33	2.60
rpoZ	*SMU_479*	RNA polymerase-associated protein RpoZ, omega subunit	376.22	35.73	0.09
	SMU_124	Putative transcriptional regulator (MarR family)	1401.42	131	0.09
	SMU_144c	Putative transcriptional regulator	204.37	84.42	0.41
glnR	*SMU_363*	Transcriptional regulator; glutamine synthetase repressor	689.41	164.60	0.24
	SMU_405c	Putative transcriptional regulator	43.42	7.36	0.17
copY	*SMU_424*	Negative transcriptional regulator, CopY	326.29	64.67	0.20
	SMU_491	Putative DeoR-type transcriptional regulator	99.42	13.66	0.14
	SMU_507	Putative transcriptional regulator (DeoR family)	5.28	1.5	0.28
	SMU_526c	Putative transcriptional regulator	21.30	68.39	3.21
	SMU_787	Putative transcriptional regulator	21.68	45.68	2.11
	SMU_921	Putative transcriptional regulator	113.49	45.14	0.40
	SMU_1012c	Putative transcriptional regulator	486.63	153.68	0.32
malR	*SMU_1566*	Putative maltose operon transcriptional repressor	30.05	3.77	0.13
celR	*SMU_1599*	Putative transcriptional regulator; possible antiterminator	88.26	230.52	2.61
	SMU_2060	Putative transcriptional regulator (LysR family)	84.76	27.61	0.33
	SMU_2108c	Putative transcriptional regulator	4.66	14.67	3.15
asnS	*SMU_1311*	Putative asparaginyl-tRNA synthetase	130.95	263.28	2.01
syfB	*SMU_1510*	Putative phenylalanyl-tRNA synthetase (beta subunit)	29.18	98.63	3.38
syv	*SMU_1770*	Putative valyl-tRNA synthetase	332.25	670.62	2.02
gatB	*SMU_1819*	Putative glutamyl-tRNA (Gln) amidotransferase subunit B	209.22	464.11	2.22
	SMU_1820c	Putative glutamyl-tRNA(Gln) amidotransferase A subunit	108.35	223.75	2.07
	SMU_1821c	Putative glutamyl-tRNA (Gln) amidotransferase subunit C	11.79	0.89	0.08
argS	*SMU_2098*	Putative arginyl-tRNA synthase	112.84	48.59	0.43
aspS	*SMU_2101*	Aspartyl-tRNA synthetase	69.30	26.21	0.38

hisS	*SMU_2102*	Histidyl-tRNA synthetase (histidine--tRNA ligase)	177.70	81.03	0.46
sygA	*SMU_445*	Putative glycyl-tRNA synthetase (alpha subunit)	28.75	66.77	2.32
	SMU_558	Isoleucine-tRNA synthetase	32.26	64.77	2.01
	SMU_773c	Lysyl-tRNA synthetase	79.49	255.33	3.21
papS	*SMU_901*	Putative poly(A) polymerase	17.73	5.08	0.29
rl19	*SMU_1288*	50S ribosomal protein L19	867.50	396.53	0.46
rl1	*SMU_1626*	50S ribosomal protein L1	25.01	94.01	3.76
rs18	*SMU_1858*	30S ribosomal protein S18	143.06	39.00	0.27
rs11	*SMU_2002*	30S ribosomal protein S11	54.52	117.89	2.16
rs13	*SMU_2003*	30S ribosomal protein S13	5.34	26.31	4.93
rl15	*SMU_2007*	50S ribosomal protein L15	22.33	98.73	4.42
rl30	*SMU_2008*	50S ribosomal protein L30	161.10	543.79	3.38
rs5	*SMU_2009*	30S ribosomal protein S5	818.27	2479.80	3.03
rl18	*SMU_2010*	50S ribosomal protein L18	1783.76	9480.21	5.31
rl6	*SMU_2011*	50S ribosomal protein L6 (BL10)	201.43	442.45	2.20
rs8	*SMU_2012*	30S ribosomal protein S8	10.00	47.32	4.73
rs14	*SMU_2014*	30S ribosomal protein S14	301.35	1073.42	3.56
rl5	*SMU_2015*	50S ribosomal protein L5	507.55	1533.15	3.02
rl29	*SMU_2019*	50s ribosomal protein L29	18.96	138.14	7.29
rl16	*SMU_2020*	50S ribosomal protein L16	106.84	255.55	2.39
rs3	*SMU_2021*	30S ribosomal protein S3	171.96	592.59	3.45
rl22	*SMU_2022*	50S ribosomal protein L22	41.21	112.91	2.74
rl4	*SMU_2024c*	50S ribosomal protein L4	198.39	553.46	2.79
rl9	*SMU_2139c*	50S ribosomal protein L9	2.17	5.99	2.76
rl23	*SMU_2166*	50S Ribosomal Protein L23	6.52	160.36	24.61
rl2	*SMU_2167*	50S Ribosomal Protein L2	90.76	244.76	2.70
rl20	*SMU_699*	50S ribosomal protein L20	41.57	4.68	0.11
rl10	*SMU_957*	50S ribosomal protein L10	3.95	23.58	5.97

grpE	*SMU_81*	Heat shock protein GrpE	205.59	64.58	0.31
	SMU_421	Translation initiation factor 2	171.56	584.76	3.41
if1	*SMU_2004*	Putative translation initiation factor IF-1	68.82	141.50	2.06
clpX	*SMU_949*	ATP-dependent protease Clp, ATPase subunit ClpX	55.82	15.16	0.27
clpP	*SMU_1672*	Putative ATP-dependent Clp protease, proteolytic subunit	77.25	17.33	0.22
htrA	*SMU_2164*	Serine protease HtrA	38.35	15.25	0.40
clpB	*SMU_1425*	Putative Clp proteinase, ATP-binding subunit ClpB	504.46	202.74	0.40
nylA	*SMU_1218*	Putative amidase	441.92	885.06	2.00
proC	*SMU_1974*	Putative pyrroline carboxylate reductase	69.24	161.26	2.33
proB	*SMU_449*	Putative gamma-glutamyl kinase	12.16	0.66	0.05
	SMU_1721c	Putative diaminopimelate decarboxylase	26.86	7.45	0.28
	SMU_24	Putative amino acid aminotransferase	57.89	17.60	0.30
	SMU_318	Putative hippurate hydrolase	105.50	430.00	4.08
	SMU_666	Putative N-acetylornithine aminotransferase	0.93	5.38	5.77
	SMU_334	Argininosuccinate synthase (citrulline-asparate ligase)	23.28	5.10	0.22
glnA	*SMU_364*	Glutamine synthetase type 1	1705.70	844.48	0.50
argC	*SMU_663*	N-acetyl-glutamate-gamma-semialdehyde dehydrogenase	0.93	5.26	5.63
	SMU_913	Putative NADP-specific glutamate dehydrogenase	31.00	71.60	2.31
pdhD	*SMU_1424*	Putative dihydrolipoamide dehydrogenase	1123.70	460.40	0.41
hisC	*SMU_1273*	Putative histidinol-phosphate aminotransferase	18.74	44.02	2.35
adhE	*SMU_148*	Putative alcohol-acetaldehyde dehydrogenase	2369.68	4846.91	2.05
hisF	*SMU_1264*	Putative imidazoleglycerol-phosphate synthase, cyclase subunit	25.60	61.23	2.39
hisA	*SMU_1265*	Putative phosphoribosyl formimino-5-aminoimidazole carboxamide ribonucleotide isomerase	37.81	134.08	3.55
hisH	*SMU_1266*	Putative glutamine amidotransferase HisH	43.85	94.21	2.15
hisG	*SMU_1271*	Putative ATP phosphoribosyltransferase	1.6	74.44	46.53
	SMU_1040c	Putative oxidoreductase, short-chain dehydrogenase/reductase	232.88	102.13	0.44
trpA	*SMU_538*	Putative tryptophan synthase, alpha subunit	12.62	43.64	3.46

pmgY	*SMU_596*	Phosphoglyceromutase	177.12	400.79	2.26
thrC	*SMU_70*	Putative threonine synthase	61.62	17.57	0.29
	SMU_966	Hhomoserine kinase	361.57	149.57	0.41
cysD	*SMU_1173*	Putative O-acetylhomoserine sulfhydrylase	111.99	365.63	3.27
cysE	*SMU_157*	Putative serine acetyltransferase; serine O-acetyltransferase	128.09	63.86	0.50
metK	*SMU_1573*	Putative S-adenosylmethionine synthetase	25.70	53.60	2.09
pfs	*SMU_1632*	Putative MTA/SAH nucleosidase	22.19	3.45	0.16
metC	*SMU_1674*	Putative aminotransferase; probable beta-cystathionase	5.04	13.95	2.77
cysK	*SMU_496*	Putative cysteine synthetase A; O-acetylserine lyase	56.86	217.99	3.83
	SMU_952	Putative methyltransferase	9.53	28.22	2.96
glmS	*SMU_1187*	Glucosamine-fructose-6-phosphate aminotransferase	121.34	53.63	0.44
gltB	*SMU_366*	NADPH-dependent glutamate synthase (small subunit)	139.32	68.98	0.50
	SMU_54	Putative amino acid recemase	32.01	66.33	2.07
leuD	*SMU_1381*	Putative 3-isopropylmalate dehydratase, small subunit	8.72	24.44	2.80
leuB	*SMU_1383*	Putative 3-isopropylmalate dehydrogenase	110.92	276.38	2.49
ilvH	*SMU_232*	Acetolactate synthase, small subunit	1.03	5.88	5.69
trpE	*SMU_532*	Putative anthranilate synthase, alpha subunit	5.25	11.17	2.13
	SMU_781	Putative prephenate dehydrogenase	296.12	996.30	3.36

Table S4 Detailed information of DEGs involved in DNA replication, repair, recombination, and oxidative stress response

Gene name	Gene name of UA159	Product	RPKM of X1	RPKM of X2	Fold change
dnaG	*SMU_821*	Putative DNA primase	31.60	14.41	0.46
dnaN	*SMU_02*	Putative DNA polymerase III, beta subunit	9.00	40.71	4.52
holB	*SMU_1662*	Putative DNA polymerase III, delta subunit	209.77	88.84	0.42
dnaB	*SMU_1922*	Putative chromosome replication protein	16.88	1.91	0.11
gyrA	*SMU_1114*	DNA gyrase A subunit	320.22	155.83	0.49
gyrB	*SMU_1277*	Putative DNA gyrase subunit B	108.53	49.01	0.45
ung	*SMU_1215*	Putative uracil DNA glycosylase	415.73	110.84	0.27
fpg	*SMU_1614*	Putative formamidopyrimidine-DNA glycosylase	186.73	52.89	0.28
mutY	*SMU_1865*	Putative A/G-specific DNA glycosylase	76.14	35.33	0.46
tagI	*SMU_2087*	Putative 3-methyl-adenine DNA glycosylase I	15.49	0.84	0.05
recA	*SMU_2085*	Recombination protein RecA	47.95	9.28	0.19
recF	*SMU_2156*	Putative RecF protein, ATPase involved in DNA repair	59.81	9.06	0.15
priA	*SMU_480*	Primosomal replication factor Y (primosomal protein N')	29.90	5.92	0.20
recM	*SMU_598*	Putative recombination protein RecM	274.96	940.68	3.42
ruvB	*SMU_64*	Holliday junction DNA helicase RuvB	81.85	25.84	0.32
	SMU_581	Putative exodeoxyribonuclease VII, small subunit	7.12	37.18	4.46
	SMU_1485c	Putative endonuclease	22.01	9.89	0.45
trxB	*SMU_463*	Putative thioredoxin reductase (NADPH)	88.47	32.87	0.37
dpr	*SMU_540*	Peroxide resistance protein Dpr	5161.90	684.00	0.13
sod	*SMU_629*	Putative manganese-type superoxide dismutase, Fe/Mn-SOD	839.00	80.16	0.10
ahpC	*SMU_764*	Alkyl hydroperoxide reductase	502.10	120.83	0.24
adhA	*SMU_127*	Putative acetoin dehydrogenase (TPP-dependent), E1 component alpha subunit	254.85	27.61	0.11
adhB	*SMU_128*	Putative acetoin dehydrogenase, E1 component beta subunit	1116.35	369.46	0.33
lplA	*SMU_131*	Putative lipoate-protein ligase	463.17	115.88	0.25
pflA	*SMU_1692*	Pyruvate-formate lyase activating enzyme	241.73	21.65	0.09

pflC	*SMU_490*	Putative pyruvate formate-lyase activating enzyme	57.91	8.94	0.15
